# A unique metabolic gene cluster regulates lactose and galactose metabolism in the yeast *Candida intermedia*

**DOI:** 10.1128/aem.01135-24

**Published:** 2024-09-06

**Authors:** Kameshwara V. R. Peri, Le Yuan, Fábio Faria Oliveira, Karl Persson, Hanna D. Alalam, Lisbeth Olsson, Johan Larsbrink, Eduard J. Kerkhoven, Cecilia Geijer

**Affiliations:** 1 Department of Life Sciences, Chalmers University of Technology, Gothenburg, Sweden; 2 Wallenberg Wood Science Center, Chalmers University of Technology, Gothenburg, Sweden; 3 Novo Nordisk Foundation Center for Biosustainability, Technical University of Denmark, Kongens Lyngby, Denmark; 4 SciLifeLab, Chalmers University of Technology, Gothenburg, Sweden; Anses, Maisons-Alfort Laboratory for Food Safety, Maisons-Alfort, France

**Keywords:** transcriptional regulation, galactose regulatory system, evolution, metabolism, non-conventional yeast, cheese whey

## Abstract

Lactose assimilation is a relatively rare trait in yeasts, and *Kluyveromyces* yeast species have long served as model organisms for studying lactose metabolism. Meanwhile, the metabolic strategies of most other lactose-assimilating yeasts remain unknown. In this work, we have elucidated the genetic determinants of the superior lactose-growing yeast *Candida intermedia*. Through genomic and transcriptomic analyses, we identified three interdependent gene clusters responsible for the metabolism of lactose and its hydrolysis product galactose: the conserved *LAC* cluster (*LAC12*, *LAC4*) for lactose uptake and hydrolysis, the conserved *GAL* cluster (*GAL1*, *GAL7*, and *GAL10*) for galactose catabolism through the Leloir pathway, and a “*GALLAC*” cluster containing the transcriptional activator gene *LAC9*, second copies of *GAL1* and *GAL10*, and a *XYL1* gene encoding an aldose reductase involved in carbon overflow metabolism. Bioinformatic analysis suggests that the *GALLAC* cluster is unique to *C. intermedia* and has evolved through gene duplication and divergence, and deletion mutant phenotyping proved that the cluster is indispensable for *C. intermedia’s* growth on lactose and galactose. We also show that the regulatory network in *C. intermedia*, governed by Lac9 and Gal1 from the *GALLAC* cluster, differs significantly from the galactose and lactose regulons in *Saccharomyces cerevisiae*, *Kluyveromyces lactis*, and *Candida albicans*. Moreover, although lactose and galactose metabolism are closely linked in *C. intermedia*, our results also point to important regulatory differences.

**IMPORTANCE:**

This study paves the way to a better understanding of lactose and galactose metabolism in the non-conventional yeast *C. intermedia*. Notably, the unique *GALLAC* cluster represents a new, interesting example of metabolic network rewiring and likely helps to explain how *C. intermedia* has evolved into an efficient lactose-assimilating yeast. With the Leloir pathway of budding yeasts acting like a model system for understanding the function, evolution, and regulation of eukaryotic metabolism, this work provides new evolutionary insights into yeast metabolic pathways and regulatory networks. In extension, the results will facilitate future development and use of *C. intermedia* as a cell-factory for conversion of lactose-rich whey into value-added products.

## INTRODUCTION

Assimilation of lactose is a rather uncommon characteristic among microorganisms, including yeasts. Growth screening of 332 genome-sequenced yeasts from the *Ascomycota* phylum showed that only 24 (<10%) species could grow on lactose, and these lactose utilizers are scattered throughout the phylogenetic tree (1). The “dairy yeasts” from the *Kluyveromyces* genus, including *Kluyveromyces lacti*s and *Kluyveromyces marxianus*, have been carefully characterized (2–5), whereas most other lactose-metabolizing yeast species remain largely understudied. Elucidating the mechanisms behind their lactose metabolism can help shed light on how eukaryotic metabolic pathways and the associated regulatory networks have evolved. Moreover, it can enable the development of new yeast species as cell factories for conversion of lactose in the abundant industrial side stream cheese whey into a range of different products (6).

Lactose, a disaccharide of D-glucose and D-galactose connected through a β-1,4-glycosidic linkage, is hydrolysed by lactase enzymes, typically β-galactosidases. Several different enzyme families encode lactases, which can be found intracellularly or extracellularly. In *Kluyveromyces* yeasts, lactose is transported across the plasma membrane by a *LAC12*-encoded lactose permease and hydrolyzed intracellularly by a *LAC4*-encoded *β*-galactosidase (5). The lactose-derived glucose and galactose moieties are further catabolized through glycolysis and the Leloir pathway, respectively. The Leloir pathway is carried out by Gal1, Gal7, and Gal10 and starts by conversion of β-D-galactose into α-D-galactose by the mutarotase domain of Gal10 (aldose-1-epimerase). Gal1 (galactokinase) then phosphorylates α-D-galactose into α-D-galactose-1-phosphate, whereafter Gal7 (galactose-1-phosphate uridylyl transferase) transfers uridine diphosphate (UDP) from UDP-α-D-glucose-1-phosphate to α-D-galactose-1-phosphate (7). The epimerase (UDP-galactose-4-epimerase) domain of Gal10 catalyzes the final step, where UDP-α-D-galactose-1-phosphate is converted to UDP-α-D-glucose-1-phosphate (8–10). In parallel to the Leloir pathway, some yeasts and filamentous fungi have an alternative galactose catabolic pathway called the oxidoreductive pathway, where galactose is first converted into galactitol through the action of an aldose reductase (9, 10).

Comparative genomic studies have revealed that the *GAL1*, *7*, and *10* genes often form a “*GAL* cluster” in yeast genomes (11). Similarly, *LAC4* and *LAC12* form a “*LAC* cluster” in, for example, *K. marxianus* and *K. lactis* (5, 11). Such metabolic gene clusters are particularly prevalent for pathways involved in sugar and nutrient acquisition and synthesis of vitamins and secondary metabolites (12). Like bacterial operons, the eukaryotic cluster genes are co-regulated in response to environmental changes, allowing the microorganism to rapidly adapt to environmental cues and avoiding deleterious recombination events and high concentrations of local protein products. For example, co-regulation of the *GAL* genes prevents accumulation of the toxic intermediate galactose-1-phosphate (11, 13).

In *Saccharomyces cerevisiae*, the three proteins *Sc*Gal4, *Sc*Gal80, and *Sc*Gal3 are responsible for galactose regulation. In the absence of galactose, the transcriptional activation domain of *Sc*Gal4 is bound to the inhibitor *Sc*Gal80. In the presence of galactose, *Sc*Gal3 relieves *Sc*Gal4 from *Sc*Gal80 in a galactose- and ATP-dependent manner, resulting in the induction of the *GAL* structural genes (14–17). Like for *S. cerevisiae*, the *K. lactis GAL* regulatory system relies on relieving *Kl*Lac9 (ortholog of *Sc*Gal4) from *Kl*Gal80 inhibition. However, *K. lactis* lacks Gal3 and instead uses a bifunctional galactokinase *Kl*Gal1 to induce both galactose and lactose genes (18). Similar to *K. lactis*, *Candida albicans* lacks Gal3 but possesses a *Ca*Gal1 with both enzymatic and regulatory functions, but in this yeast, the *GAL* gene expression is controlled by transcription factors *Ca*Rtg1/*Ca*Rtg3 (19) and/or *Ca*Rep1/*Ca*Cga1 (14, 20). Such transcriptional rewiring is common among yeasts, which calls for coupling of comparative genomics with detailed mutant phenotyping and transcriptional analysis to decipher how regulation occurs in individual species.

While galactose and lactose metabolism in *S. cerevisiae* and *K. lactis* has long served as a model system for understanding the function, evolution, and regulation of eukaryotic metabolic pathways, the corresponding knowledge in other yeasts is scarce. One such understudied species is *Candida intermedia*, a haploid yeast belonging to the *Metschnikowia* family in the CUG-Ser1 clade (1), which has previously received attention as a fast-growing yeast on xylose (21–26). *C. intermedia* is one of very few yeasts in the *Metschnikowia* family that can grow on lactose (1), and it has been used for cheese whey bioremediation in the past (27). Our previous works on the in-house isolated *C. intermedia* strain CBS 141442 in terms of genomics, transcriptomics, and physiology (24, 28, 29) and genetic toolbox development (30) provide a stable platform for exploration of the genetic determinants of lactose metabolism in this yeast. In the present study, we show that *C. intermedia* possesses a unique “*GALLAC*” cluster, in addition to the conserved *GAL* and *LAC* clusters, which proved to be essential for growth on lactose and highly important for growth on galactose. Characterization of the individual *GALLAC* cluster genes revealed differentiation in their functionality, enabling the yeast to regulate the expression of galactose and lactose genes differently. This cluster represents a new, interesting example of metabolic network rewiring in yeast and sheds light on how *C. intermedia* has evolved into an efficient lactose-assimilating yeast.

## RESULTS

### *C. intermedia* is among the top five lactose growers out of 332 sequenced ascomycetous yeasts

**Fig 1:**
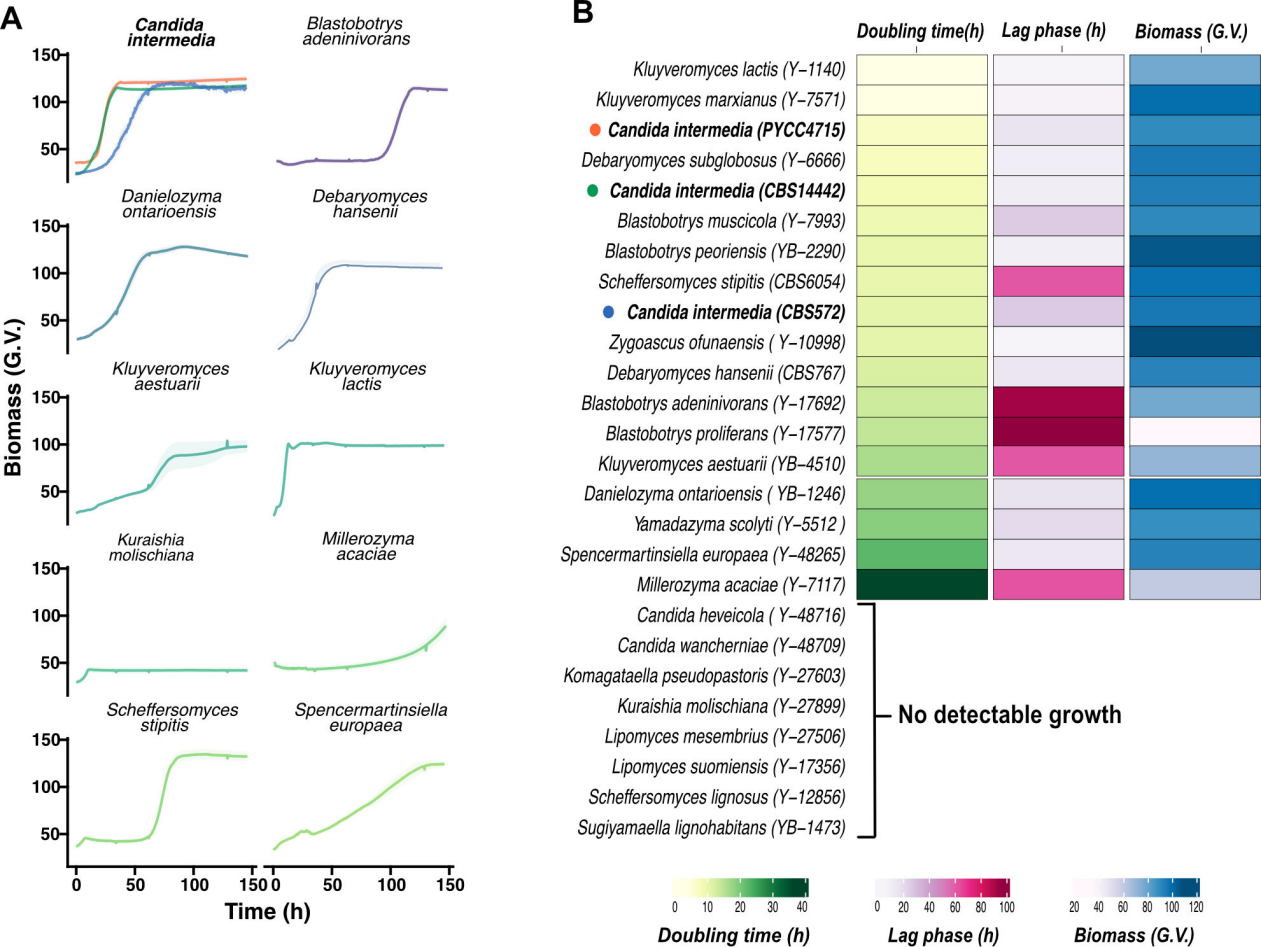
*Candida intermedia* is one of the top five fastest lactose-growing yeast species. (**A**) Representative growth profiles of 10/24 lactose-yeast species including three different *C. intermedia* strains that are depicted in different colors. The graphs depict data procured using a GrowthProfiler in 96-well format, represented as mean ± standard deviation (shaded region) for biological triplicates. On the y-axis, yeast biomass is depicted in green values (G.V.—corresponding to growth based on pixel counts, as determined by GrowthProfiler) and is plotted against time (h) on the x-axis. (**B**) Heat map showing doubling time (h), lag phase duration (h), and final biomass (G.V.) measured for all the tested strains in minimal media containing lactose as the sole carbon source and plotted as an average of three biological replicates. Strains are ranked based on their doubling time, from low to high.

As a start, we wanted to assess the capacity of *C. intermedia* to grow on lactose compared with other yeasts. We cultured the 24 out of 332 ascomycetous species that have previously scored positive for lactose growth (1), as well as *C. intermedia* strains CBS 572 (type strain), CBS 141442, and PYCC 4715 (previously characterized for utilization of xylose) (1, 25). The yeasts displayed different growth patterns in lag phase, doubling time, and final biomass (Fig. 1; Fig. S1). When ranked based on lowest doubling time, *K. lactis* and *K. marxianus* were the fastest growers on lactose, closely followed by *C. intermedia* strains PYCC 4715 and CBS 141442, *Debaryomyces subglobusus*, and *Blastobotrys muscicola* (Fig. 1; Fig. S1). Species such as *Kluyveromyces aestuarii*, *Millerozyma acaciae*, and *Lipomyces mesembris* showed poor or no growth under the conditions tested, while others had very long lag phases. Thus, under the assessed conditions, our results establish *Candida intermedia* as one of the top five fastest lactose-growing species out of the 24 ascomycetous yeasts tested.

### Genomic and transcriptomic analyses identify three gene clusters involved in lactose and galactose assimilation

To identify the genetic determinants for lactose metabolism in *C. intermedia* CBS 141442, we searched the genome for orthologs of known genes involved in the uptake and conversion of lactose and its tightly coupled hydrolysis-product, galactose. We found several genes encoding expected transcription factor orthologs including *LAC9*, *GAL4*, *RTG1*, *RTG3*, *REP1*, and *CGA1* that have been associated with lactose and galactose metabolism in *K. lactis* (31), *S. cerevisiae* (32), and *C. albicans* (19, 20). In accordance with previous reports for yeasts belonging to the genus *Candida* (8), we did not find orthologs of *GAL80*, strongly suggesting that *C. intermedia* does not possess the Gal3-Gal80-Gal4 regulon.

**Fig 2:**
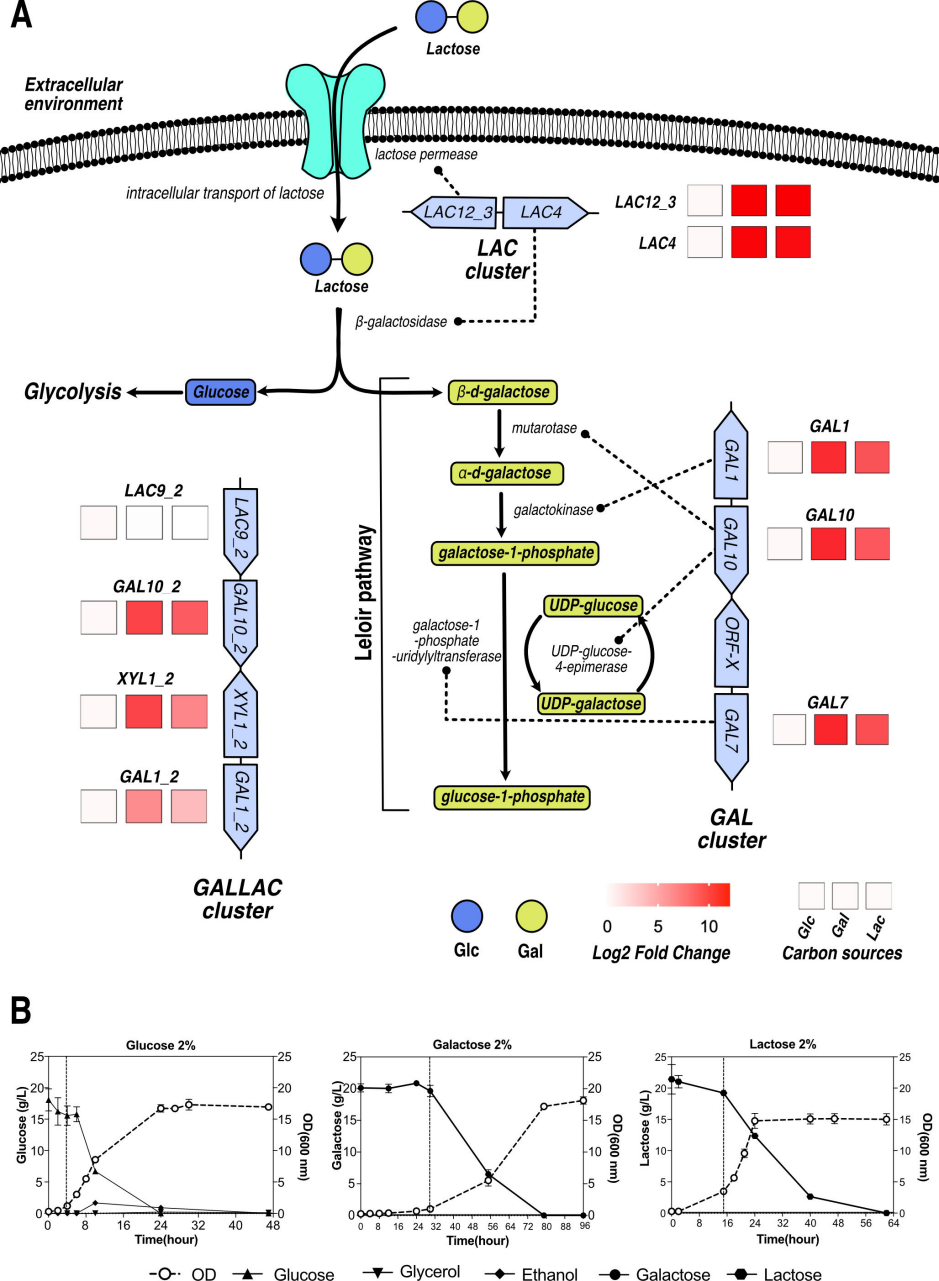
Genomic and transcriptomic analyses identified three gene clusters involved in lactose and galactose assimilation. (A) A schematic representation of lactose and galactose metabolic pathways and results of RNA-sequencing (RNA-seq) data analysis showing expression of different genes (as present in clusters) upregulated in galactose or lactose compared with glucose. Lactose uptake and transport into the cell are enabled by the *LAC12_3*-encoded lactose permease followed by hydrolysis to glucose (blue circle: Glc) and galactose (yellow circle: Gal) enabled by *LAC4*-encoded β-galactosidase enzyme. Glucose is further metabolized via glycolysis. Galactose is metabolized via the Leloir pathway, encoded by three clustered genes, *GAL1* (galactokinase), *GAL7* (galactose-1-phosphate-uridylyltransferase), and *GAL10* (mutarotase and UDP-glucose-4-epimerase). The enzymatic functions for the genes are depicted by dotted lines based on genome sequence data for *C. intermedia* CBS 141442. Legend shows log2 fold change with carbon sources tested represented as Glc for 2% glucose-, Gal for 2% galactose-, and Lac for 2% lactose-containing media. Gene expression log fold change is normalized with glucose as control. (B) Bioreactor profiles of *C. intermedia* CBS 141442 growth in minimal media containing either 2% glucose, galactose, or lactose with sampling timepoints for RNA-seq analysis depicted by the dotted line for each carbon source. Individual plots show glucose, galactose, or lactose on left y-axis and biomass [optical density measured at 600 nm (OD_600_)] on the right y-axis against time (hours) on the x-axis. Data are represented as mean ± standard deviation (error bars).

Moreover, the genome of *C. intermedia* contains the conserved *GAL* cluster including *GAL1*, *7*, and *10* genes as well as an *ORF-X* gene encoding a putative glucose-4,6-dehydratase, similar to *GAL* clusters in *Candida/Schizosaccharomyces* strains (8, 11) (Fig. 2A). We also identified the conserved *LAC* cluster containing the *β*-galactosidase gene *LAC4* and lactose permease gene *LAC12* (2, 3, 5), which correlates well with *C. intermedia* predominantly displaying intracellular *β*-galactosidase activity (data not shown). To our surprise, *C. intermedia* also possesses a third cluster, hereafter referred to as the *GALLAC* cluster, containing a putative transcriptional regulator gene *LAC9* (*LAC9_2*) next to a second copy of the *GAL1* gene (*GAL1_2*), followed by one of the three xylose/aldose reductase genes (*XYL1_2*) previously characterized in *C. intermedia* (28), and lastly, a second copy of *GAL10* (*GAL10_2*). Interestingly, the *GAL10_2* gene is shorter than *GAL10* in the *GAL* cluster and seems to encode only the epimerase domain, similar to *GAL10* orthologs in *Schizosaccharomyces* species and filamentous fungi (8).

Next, we performed transcriptome analysis using RNA-seq technology on the CBS 141442 strain cultivated in media containing 2% of either lactose, galactose, or glucose (Fig. 2B). Compared with glucose, we identified 516 genes with over twofold differential expression in galactose: 58 downregulated and 458 upregulated. In lactose, 423 genes were differentially expressed: 56 downregulated and 367 upregulated. Notably, 299 genes were >twofold differentially expressed in both galactose and lactose, and the vast majority of the genes were regulated in the same direction, suggesting a strong regulatory and metabolic interconnection between the two carbon sources (Table S1). All genes in the *LAC* and *GAL* clusters and the *GAL10_2* and *XYL1_2* genes in the *GALLAC* cluster were among the 20 highest upregulated genes in both galactose and lactose (Table S1). Also *GAL1_2* was greater than twofold upregulated on both of these carbon sources, while the *LAC9_2* gene was constitutively expressed in all three carbon sources (Fig. 2A). Among the most highly expressed genes, we also identified several additional *LAC12* paralogs encoding putative disaccharide transporters, *HGT1_2* and *MAL11* that likely encode galactose and maltose transporters, and *BGLS_2* predicted to encode a β-glucosidase (Table S1). These gene products may have functions during growth on galactose and lactose, or they may simply be derepressed in the absence of glucose.

### The *GALLAC* cluster is essential for growth on lactose and unique to *C. intermedia*

**Fig 3:**
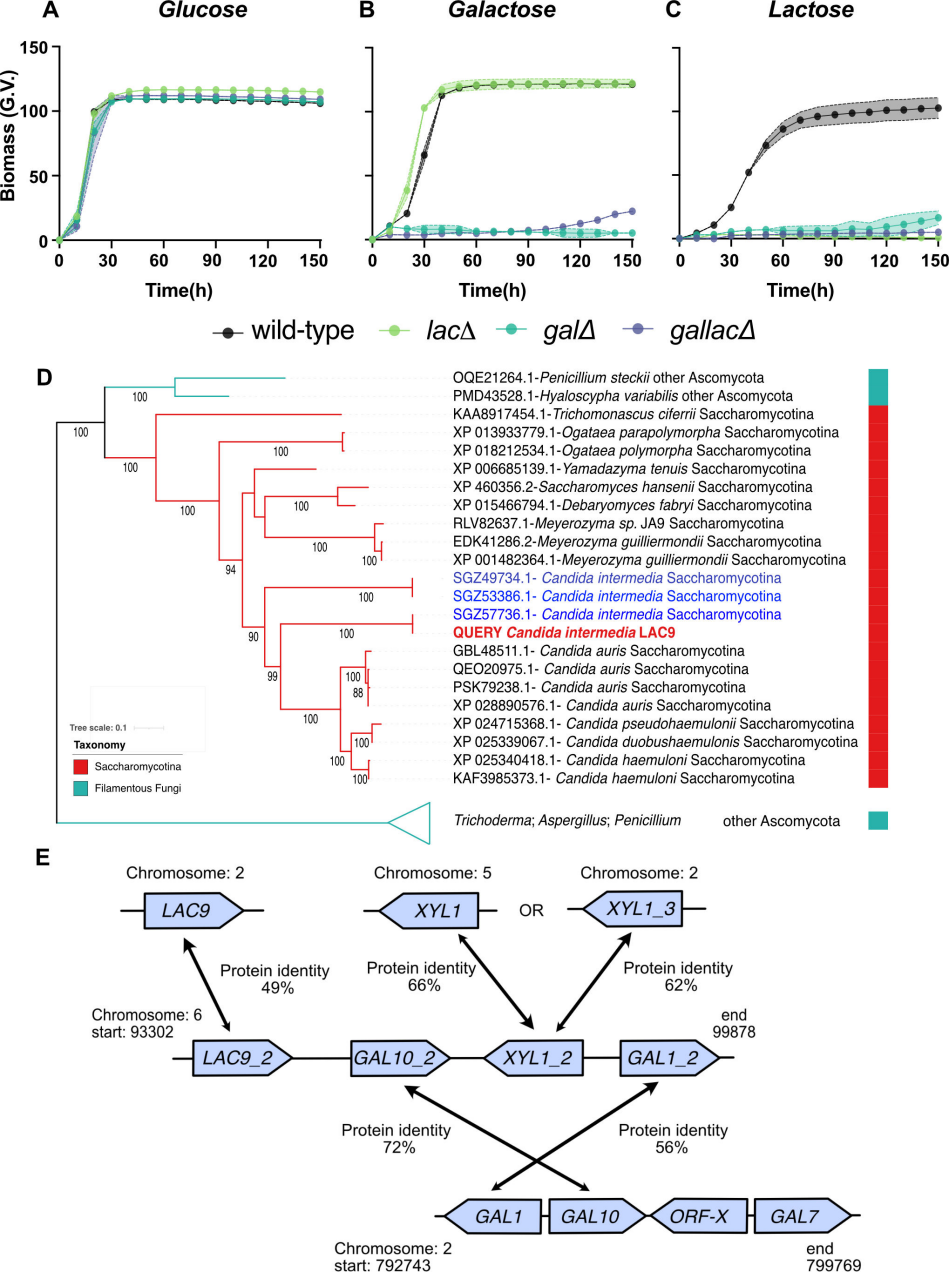
The *GALLAC* cluster is essential for growth on lactose, highly important for growth on galactose, and unique to *C. intermedia*: cluster deletion mutants of *C. intermedia* were characterized by growth on (A) glucose, (B) galactose, and (C) lactose. Legend shows the wild-type strain (black), *LAC* cluster mutant (light-green), *GAL* cluster deletion mutant (dark-green), and *GALLAC* cluster deletion mutant (purple), depicted in the graph with biomass as (G.V.) corresponding to growth based on pixel counts as determined by GrowthProfiler on the y-axis, against time (h) on the x-axis. Data are represented as mean ± standard deviation (shaded region) for biological triplicates. (D) Phylogenetic tree representing closest neighbors of *Ci*Lac9 based on protein identity across the tested 332 yeast species. Query sequence is represented in red with the names of the protein sequences followed by species name and phylum/subphylum at the end of the branch. (E) Graphical representation of genomic location of cluster and individual genes, which are paralogs to *GALLAC* cluster genes in *C. intermedia* and their respective protein identity.

To decipher the importance of the three clusters for galactose and lactose metabolism in *C. intermedia*, we deleted the clusters one by one using the split-marker technique previously developed for this yeast (30). The cluster deletion mutants (*lacΔ*, *galΔ*, and *gallacΔ*) grew almost as well as the wild-type (WT) strain in minimal media containing glucose (Fig. 3A). As expected, *galΔ* failed to grow on galactose, which can be attributed to the complete shut-down of the Leloir pathway, whereas the *lacΔ* grew like the WT. Interestingly, no growth was observed for the *gallacΔ* in galactose during the first 90 h, whereafter it started to grow slowly (Fig. 3B). With lactose as a carbon source, both *lacΔ* and *gallacΔ* completely failed to grow, whereas *galΔ* started to grow slowly after approx. 100 h (Fig. 3C). Neither of the slow-growing mutants reached the same biomass as the WT during a total of 200 h of cultivation. It is important to note that the late growth observed could be due to suppressing mutations in other parts of the genome or complementation by enzymes expressed from paralogous genes. Nevertheless, our results show that the *GALLAC* cluster is essential for growth on lactose and at least highly important for growth on galactose.

To the best of our knowledge, the existence of a *GALLAC*-like cluster and its interdependence with the *GAL* and *LAC* clusters have never been reported before. This, along with the severe growth defects of *gallacΔ*, encouraged us to determine the origin and prevalence of the cluster in other yeasts. First, we performed a comparative genomic analysis among the data set of 332 genome-sequenced ascomycetous yeasts (1), determining which yeasts contain the *GAL*, *LAC*, and *GALLAC* cluster orthologs and determining the genes’ respective genomic location. Although *GAL1* and *GAL10* were found clustered together as parts of the conserved *GAL* clusters in 150/332 species, *C. intermedia* was the only species where these genes also clustered with *LAC9* and *XYL1* genes (Table S2). Importantly, the *GALLAC* cluster was found in the genomes of all five *C. intermedia* strains assessed (CBS 572, CBS 141442, PYCC 4715, NRRL Y-981, and P5906) (Table S3). Next, to decipher the evolutionary events that led to the formation of the *GALLAC* cluster, we generated phylogenetic trees for each individual gene product of the cluster (exemplified in Fig. 3D; additional trees are shown in Fig. S2 through S4). Our analysis revealed that although the amino acid identities between the paralogs in *C. intermedia* are relatively low (56% for Gal1 and Gal1_2, 72% for Gal110 and Gal10_2, 49% for Lac9_2 and Lac9, and 66% and 62% for Xyl1_2 compared with Xyl1 and Xyl1_3, respectively); the identities between the paralogs are still higher than for most orthologs in other species. Combined, these results strongly suggest that the unique *GALLAC* cluster has evolved within *C. intermedia* through gene duplication and divergence (Fig. 3E).

### Deletion of individual genes in the *GAL* and *GALLAC* clusters reveals importance of Lac9_2 and Gal1_2 for galactose and lactose metabolism

To elucidate the physiological function of genes situated in the *GALLAC* cluster and to better understand the interdependence between the clusters, we deleted individual genes in both the *GALLAC* and *GAL* clusters. The mutant phenotypes were compared with WT and complete cluster deletions regarding growth, consumption of sugars, and production of metabolites in defined media containing either 2% galactose or lactose.

**Fig 4:**
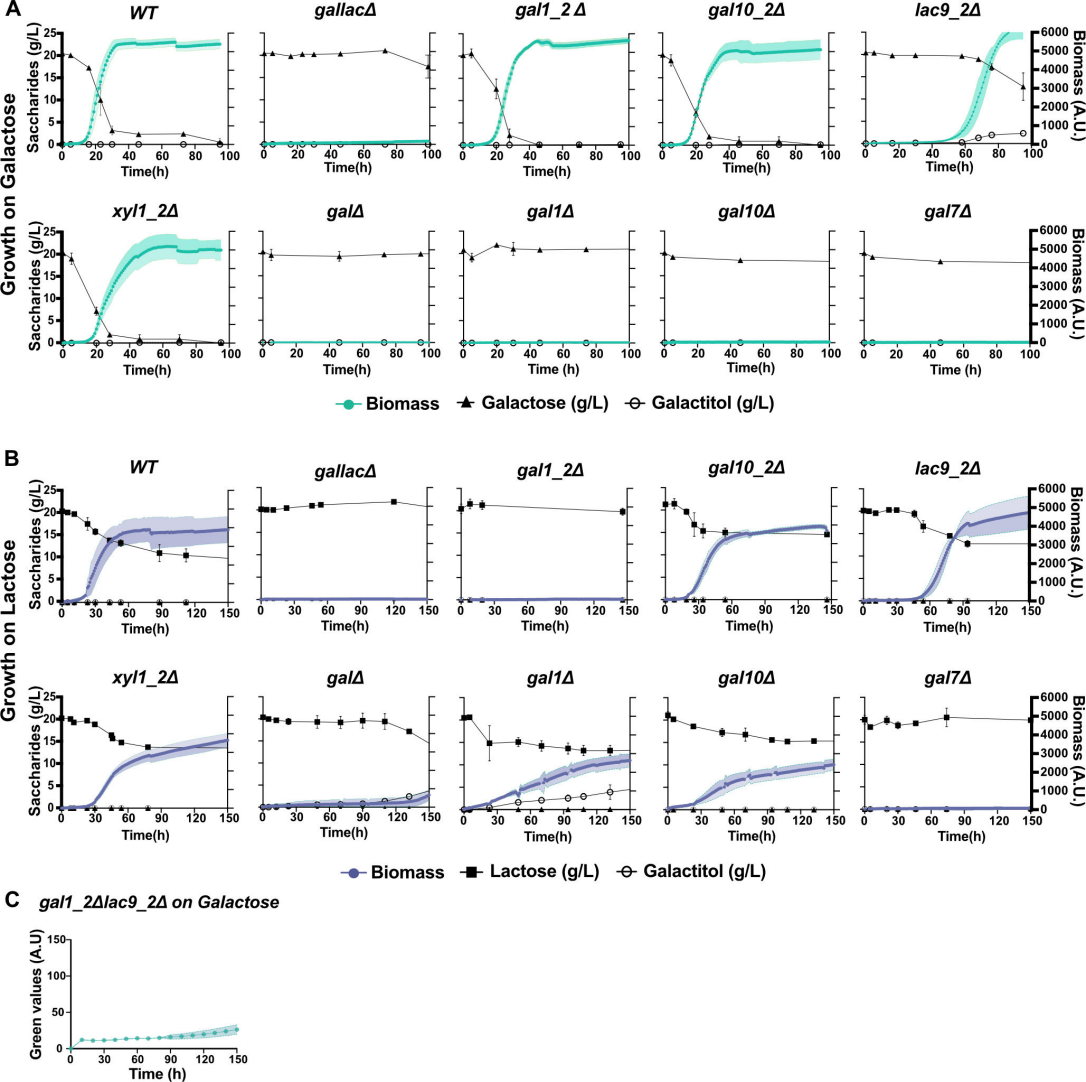
Deletion of individual genes in the *GAL* and *GALLAC* clusters reveals importance of Lac9 and Gal1_2 for galactose and lactose metabolism: Growth and metabolite profiles for deletion mutants of individual genes in the *GAL* and *GALLAC* cluster of *C. intermedia*, in (A) galactose and (B) lactose containing media in a cell growth quantifier (CGQ). Graphs represent biomass (filled circle; gal—dark green; lac—purple) on the right y-axis, consumption of respective sugars (filled triangle for galactose in g/L or filled square for lactose in g/L), and metabolite production (open circle for galactitol in g/L) on the left y-axis [depicted by saccharides (g/L)], plotted against time (h) on x-axis. Data are represented as mean ± standard deviation (shaded region for biomass and bars for sugars and metabolites) for biological triplicates. (C) Growth phenotype for *gal1_2Δlac9_2Δ* double deletion mutant (in green) in galactose. Graph shows biomass as G.V. (corresponding to growth based on pixel counts, as determined by GrowthProfiler) on the y-axis against time (h) on the x-axis. Data are represented as mean ± standard deviation (shaded region) for biological triplicates.

With galactose as carbon source, deletion of *LAC9_2* located in the *GALLAC* cluster resulted in an extended lag phase accompanied by galactitol production, indicating that this putative transcription factor is involved in the regulation of galactose metabolism (Fig. 4A). In contrast, deletion of the other genes in the *GALLAC* cluster did not result in severe growth defects. For mutants deleted of individual *GAL* cluster genes and grown in galactose, we saw the expected severe growth defects for *gal1Δ*, *gal7Δ*, and *gal10Δ* (Fig. 4A). However, *gal10Δ* repeatedly displayed some growth after a very long lag phase of approx. 250 h (Fig. S5), which could suggest that Gal10_2 from the *GALLAC* cluster can partly complement the deletion of *GAL10* from the *GAL* cluster.

With lactose as carbon source, *lac9_2Δ* displayed a delay in the onset of growth similar to what was observed with galactose, while *gal10_2Δ* and *xyl1_2Δ* grew like the WT (Fig. 4B). However, in contrast to growth on galactose, deletion of *GAL1_2* alone abolished growth and resembled the deletion of the whole *GALLAC* cluster, indicating an important function for the encoded protein in lactose metabolism and a clear phenotypic difference between the two carbon sources. On the contrary, deletion of *GAL1* from the *GAL* cluster did not fully abolish growth on lactose, but growth was slower and accompanied with substantial accumulation of galactitol (3.4 g/L from a total of 10.8 g lactose consumed), suggesting that most of the lactose-derived galactose is catabolized through the action of an aldose reductase (such as Xyl1_2), rather than through the putative galactokinase Gal1_2 in this mutant. Also, *gal10Δ* grew slowly but with no measurable accumulation of galactose or galactitol, again suggesting that the *GAL10_2* in the *GALLAC* cluster can partly complement this deletion. Deletion of the only copy of the *GAL7* gene encoding for galactose-1-phosphate uridylyltransferase resulted in complete growth inhibition on lactose (as for galactose), and we speculate that the severe growth phenotype is due to the accumulation of toxic intermediate galactose-1-phosphate as seen in *S. cerevisiae* in previous studies (13).

Since no single deletion resembled the growth defect observed for *gallacΔ* on galactose, we hypothesized that two or more genes must be deleted for the same phenotype to occur. Because construction of double mutants in *C. intermedia* is very time-consuming, we prioritized creating a *lac9_2Δgal1_2Δ* double deletion mutant, as deletion of *LAC9_2* extended the lag phase in both carbon sources and deletion of *GAL1_2* led to a severe growth defect in lactose. Indeed, the resulting strain displayed a growth defect in galactose strikingly similar to that of the complete *GALLAC* cluster mutant (Fig. 4C). Although we cannot rule out that other double mutants in the *GALLAC* cluster would also exhibit severe growth phenotypes, we can conclude that Lac9_2 and Gal1_2 have important functions during both galactose and lactose growth.

### Lac9 binding motifs are found in promoters in the *GALLAC* cluster genes and regulates their expression in galactose and lactose

**Fig 5:**
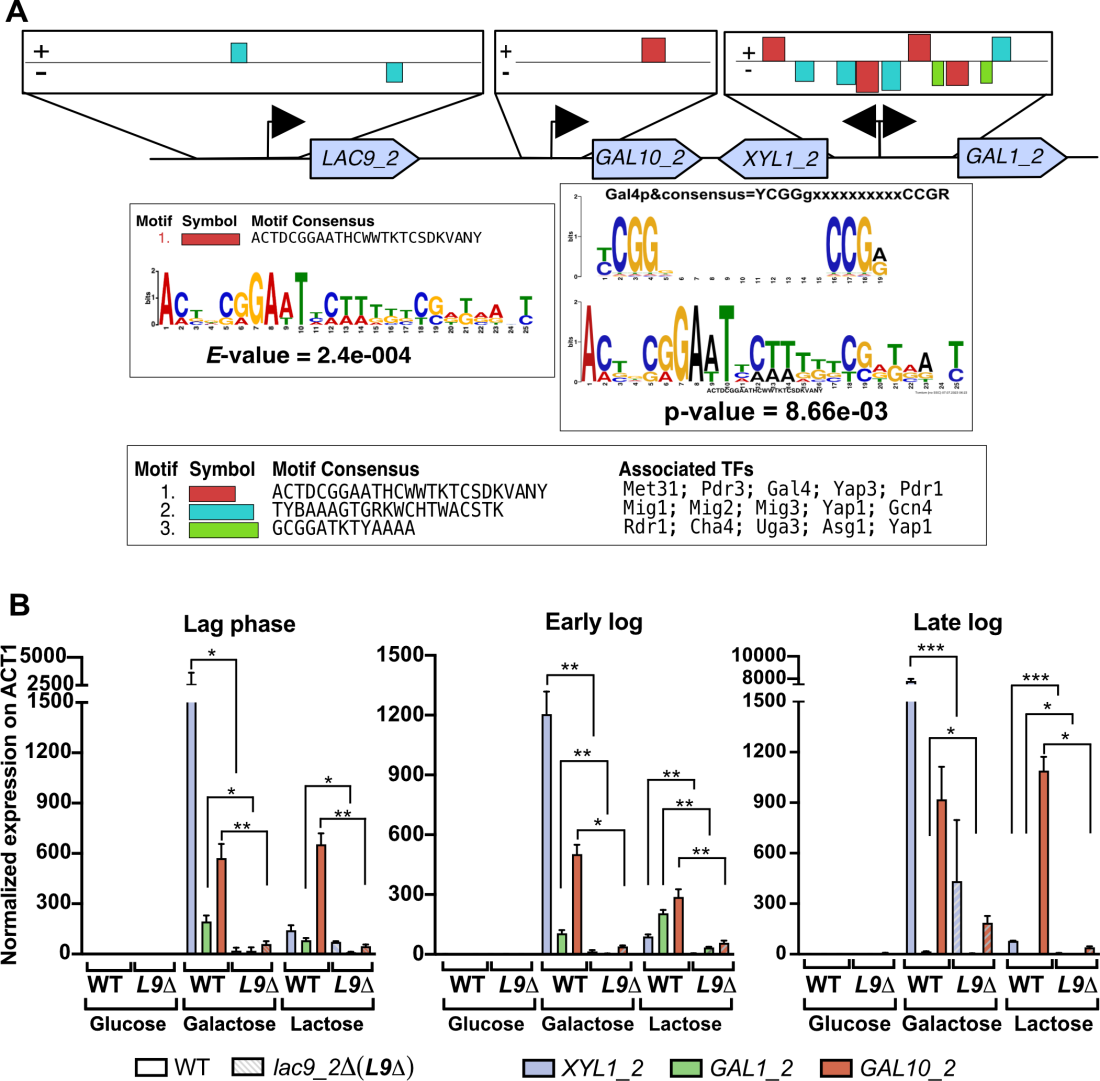
Lac9 binding motifs are found in promoters in the *GALLAC* cluster. (A) Graphical representation of results of transcription binding motif analysis for promoters of individual genes in the *GALLAC* cluster, using MEME (version 5.5.43). Locations of three statistically significant promoter binding motifs found in the promoters of the cluster genes are depicted. Motif consensus of the binding motif with the lowest *E* value score of the overall match of the motifs in the input sequence, and the Gal4/Lac9 consensus sequence and its associated *P* value, as well as a list of three (statistically significant) motifs found in the promoters of *GALLAC* cluster genes and the transcription factors associated to these motifs derived from Yeastract database using TomTom. (B) Quantitative PCR results for expression profiles of *GALLAC* cluster genes in *C. intermedia* wild type and *lac9_2Δ* grown in glucose, galactose, or lactose. Samples were taken during different growth phases: WT (glucose : lag = 5 h; early log = 10 h; late log = 20 h; galactose : lag = 5 h; early log = 24 h; late log = 44 h; and lactose : lag = 5 h; early log = 28 h; late log = 40 h) and *lac9_2Δ* (glucose : lag = 5 h; early log = 10 h; late log = 20 h; galactose : lag = 5 h; early log = 44 h; late log = 80 h; and lactose : lag = 5 h; early log = 44 h; late log = 80 h). Data are represented as mean ± standard deviation (error bars) for biological and technical triplicates. A two-tailed *t*-test was used for statistical analysis; *P < 0.05, **P < 0.01, and ***P < 0.001.

To better understand the putative role of Lac9_2 as a transcriptional regulator, we performed Multiple Em for Motif Elicitation (MEME; version 5.5.43) (33) analysis to identify conserved transcription factor binding motifs in gene promoters in the three clusters. The analysis revealed Lac9 (Gal4) binding motifs (*P* value = 8.66 × 10^−3^) in the promoters of *GAL1_2*, *XYL1_2*, and *GAL10_2* in the *GALLAC* cluster (Fig. 5A), but not in the promoters in the *GAL* and *LAC* clusters. These results confirm the bioinformatic analysis of the 332 ascomycetous yeast recently published, showing that *C. intermedia* and many other CUG-Ser1 clade yeasts lack Lac9/Gal4 binding sites in the *GAL* clusters (11). Besides Lac9/Gal4, we also found binding sites for other transcription factors, including Mig1 that is known to repress *GAL* genes in *S. cerevisiae* (34) (Fig. 5A).

To determine if the deletion of *LAC9_2* affects the expression of *GALLAC* cluster genes, we grew WT and *lac9_2Δ* in glucose, galactose, and lactose. Samples were taken during the lag phase and early and late logarithmic phases for the two strains, and quantitative PCR (qPCR) was performed to measure the expression of *GAL1_2*, *GAL10_2*, and *XYL1_2*. While both strains exhibited low expression of all three genes in glucose, the WT strain showed significant upregulation of all three genes in galactose and lactose, although *GAL1_2* was not upregulated to the same extent as the other two genes (in line with the RNA-seq results). In contrast, the *lac9_2Δ* strain had markedly lower expression of all three genes throughout growth in galactose and lactose, indicating a clear role of Lac9_2 as a regulator of the *GALLAC* cluster genes (Fig. 5B).

Besides *LAC9_2* in the *GALLAC* cluster, our comparative genomic analysis also identified a second, non-clustered *LAC9* gene (Fig. 3) as well as *GAL4* gene. All three proteins have predicted Gal4-like DNA-binding domains, but they differ substantially in protein sequence identity (45% identity between Lac9_2 and Lac9, while Gal4 is only 18% and 19% identical to Lac9 and Lac9_2, respectively). Deletion mutants of *LAC9* and *GAL4* did not display growth defects on lactose or galactose (Fig. S6). Although we cannot exclude the possibility of functional redundancy between the Lac9 paralogs, our results indicate that the *GALLAC* cluster-derived Lac9_2 is the most important transcriptional regulator for galactose and lactose metabolism in *C. intermedia* and that Lac9_2 likely exerts its regulatory role by directly binding to the promoters and controlling the expression of the *GALLAC* cluster genes.

### Gal1_2 is required for the induction of *LAC* cluster genes in *C. intermedia*

**Fig 6:**
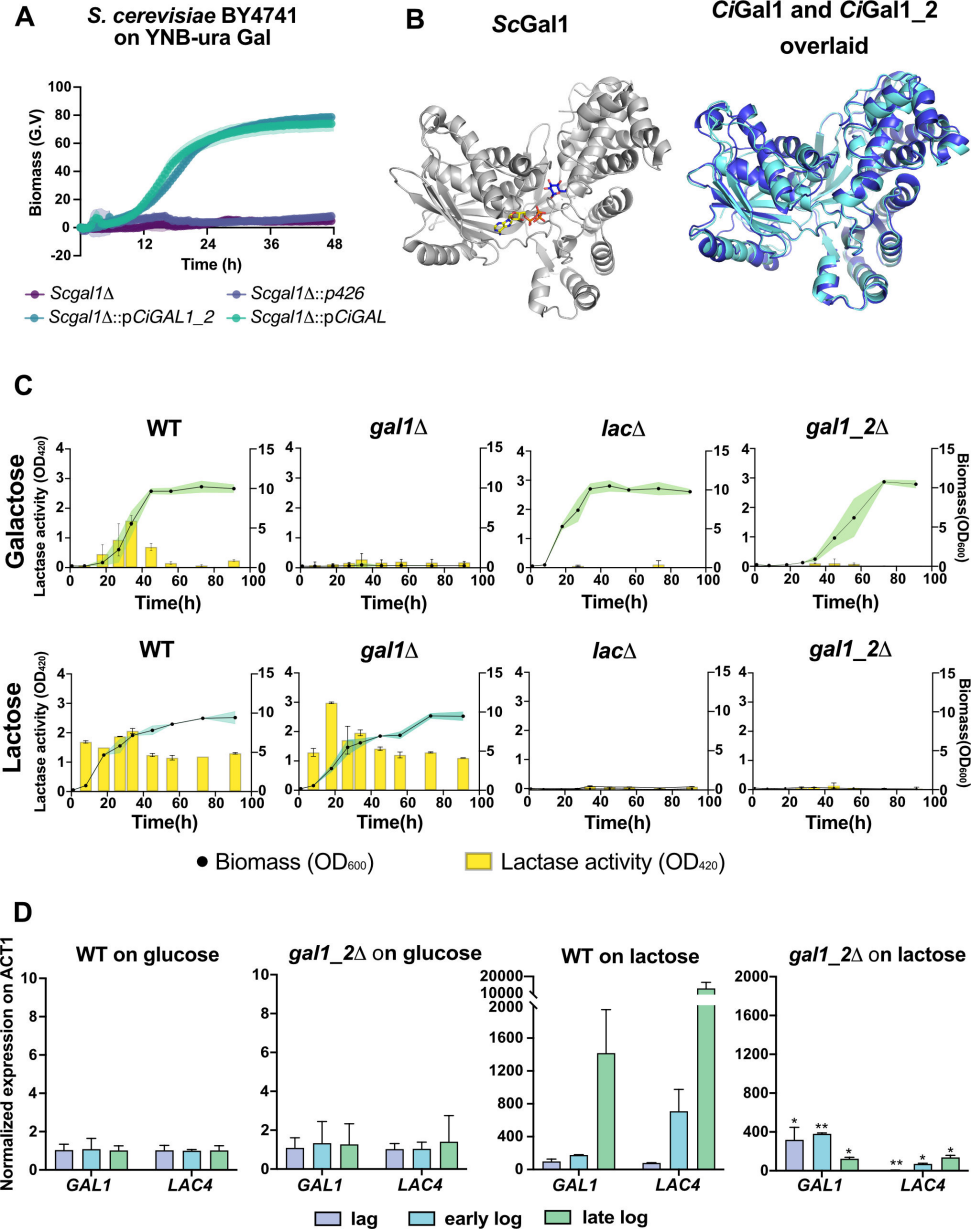
Characterization of *C. intermedia*’s Gal1 and Gal1_2 reveals important functional differences. (A) Results of complementation test of codon-optimized *CiGAL1* and *CiGAL1_2* expressed in *S. cerevisiae* BY4741 gal1*Δ* mutant. Growth profiles are depicted for *Scgal1Δ* (dark-purple), *Scgal1Δ* with plasmid p426 containing *URA3* marker (light-purple), *Scgal1Δ* with p*CiGAL1_2* (dark-green), and *Scgal1Δ* with p*CiGAL1* (light-green). Time (in hours) on x-axis is plotted against biomass (G.V.) on y-axis. Data are represented as mean ± standard deviation (shaded region for biomass) for biological triplicates. (B) Structure of *Sc*Gal1 (gray) in complex with AMPPNP and α-galactose next to the superimposed AlphaFold2-predicted structures of Gal1 (cyan) and Gal1_2 (blue) in the same orientation, showing their high structural similarity. (C) β-Galactosidase assay on galactose- and lactose-grown cultures of *C. intermedia* wild-type, *lacΔ*, *gal1Δ*, and *gal1_2Δ* strains. Graphs show lactase activity (OD_420_) plotted on left y-axis against time (in hours) on x-axis and biomass (OD_600_) plotted on right y-axis. (D) Quantitative PCR results for *LAC4* and *GAL1* gene expression in *C. intermedia* wild type and *gal1_2Δ* grown in glucose or lactose. Samples were taken during different growth phases (on glucose, lag = 5 h, early log = 10 h and late log = 20 h and on lactose, lag = 5 h, early log = 28 h, late log = 40 h, although it should be noted that *gal1_2Δ* did not grow in lactose). Data are represented as mean ± standard deviation (error bars) for biological and technical triplicates. A two-tailed *t*-test was used for statistical analysis; *P < 0.05, **P < 0.01, and ***P < 0.001.

Our deletion mutant phenotyping results suggest that Gal1 and Gal1_2 have at least partly different physiological functions in *C. intermedia* (Fig. 4). As the two *GAL1* genes are highly upregulated on both galactose and lactose in the WT strain (Fig. 2), we speculated that the encoded proteins must differ in their activities as galactokinases or regulators. To this end, we expressed both proteins in *S. cerevisiae* BY4741 *gal1Δ*, which successfully rescued the mutant’s growth defect on galactose (Fig. 6A). This experiment demonstrates that both proteins have galactokinase activity, at least when expressed in *S. cerevisiae*. We also compared the predicted structures of Gal1 and Gal1_2 using AlphaFold2 (35, 36), observing that even though the amino acid sequence identity between the two proteins is as low as 56%, the protein structures are very similar to each other (rmsd 0.490 Å; Fig. 6B) as well as to the experimentally solved structure of *Sc*Gal1^42^ (rmsd 0.778 and 0.758 Å for Gal1 and Gal1_2, respectively). Additionally, we observed that the amino acids interacting with galactose in *Sc*Gal1 (PDB ID: 2aj4) are identical to those in the *Ci*Gal1 proteins, apart from Asn213 in *Sc*Gal1 (Asn205 in *Ci*Gal1), which interacts with the O2 hydroxyl group, which in *Ci*Gal1_2 is instead a serine residue (Ser199). The active site clefts of all enzymes are only big enough to accommodate monosaccharides like galactose. Thus, it is highly unlikely that they bind to other, larger substrates such as lactose (Fig. 6B). In *S. cerevisiae*, the regulator *Sc*Gal3 is similar in structure to the galactokinase *Sc*Gal1 but has lost its galactokinase activity due to an addition of two extra amino acids (Ser-Ala dipeptide) (37). However, no such structural changes were seen for *Ci*Gal1 or *Ci*Gal1_2 that could help us predict regulatory functions.

We examined the role of Gal1 and Gal1_2 as regulators of lactose metabolism by performing β-galactosidase assays with *C. intermedia* strains *gal1Δ* , *gal1_2Δ*, and the WT (positive control) and *lacΔ* (negative control). Our RNA-seq data showed that in the WT, *LAC4* is expressed during growth in both galactose and lactose (Fig. 2). Thus, we assessed the lactase activity during growth in both these sugars to include at least one condition where all strains could grow. Lactase activity was readily detected in the WT during growth in both galactose and lactose. In *gal1Δ* cells that do not grow in galactose, we only observed lactase activity during lactose growth. In contrast, *gal1_2Δ* that only grows in galactose displayed no lactase activity during growth in this carbon source, similar to *lacΔ* where the lactase gene *LAC4* has been deleted (Fig. 6C). These results suggest that Gal1_2 is essential for induction of lactase activity.

Moreover, qPCR analysis of the WT and *gal1_2Δ* revealed that *LAC4* expression in lactose was diminished in *gal1_2Δ* as compared with the WT. Conversely, *GAL1* expression was higher in the mutant compared with the WT during the first 28 h of cultivation and was then markedly downregulated at 40 h, likely due to the lack of growth for this mutant in lactose (Fig. 6D). This indicates that regulation of *LAC4* is exerted on the transcriptional level and fortifies the growth phenotyping results where we saw a clear difference in growth on galactose (+) and lactose (−) for *gal1_2Δ*. Overall, these results firmly establish a difference in function between Gal1 and Gal1_2, where a lack of Gal1_2 diminishes lactase activity while a lack of Gal1 does not, and further indicate important differences in the regulation of lactose and galactose metabolism and growth.

## DISCUSSION

In this work, we have investigated how galactose and lactose are metabolized in the non-conventional yeast *C. intermedia* and shed light on the genetic determinants behind this trait. Interestingly, we found that the genome of *C. intermedia* contains not only the conserved *GAL* and *LAC* clusters but also a unique *GALLAC* cluster that has evolved through gene duplication and divergence. Our results show that galactose metabolism is impaired in both *galΔ* and *gallacΔ* strains, while lactose metabolism is impaired in all three cluster deletion strains. As the *GAL* cluster encodes the structural genes in the Leloir pathway, it is logical that deletion of this cluster effectively suppresses galactose metabolism. In the *gallacΔ* strain, the *GAL* cluster remains intact, but the strain failed to grow, indicating that both clusters are needed for galactose and lactose growth and thus demonstrating their interdependence. By combining results from comparative genomics, transcriptomics analysis, deletion mutant phenotyping, and metabolite profiling, we have started to unravel parts of the regulatory networks of the three clusters and can show that the *GALLAC* cluster plays a vital role in regulating both galactose and lactose metabolism in this yeast. With the Leloir pathway of budding yeasts acting like a model system for understanding the function, evolution, and regulation of eukaryotic metabolic pathways, this work adds interesting new pieces to the puzzle.

Our results show that *C. intermedia* grows relatively fast on lactose, and strains of this species have been isolated several times from lactose-rich niches including fermentation products like white-brined cheese (38) and cheese whey (39). In these lactose-rich environments, survival likely necessitates a genetic makeup that can help outcompete rivaling microorganisms. Is the *GALLAC* cluster facilitating the fast lactose growth observed for *C. intermedia*, and if so, how? This is currently unresolved, but the genes within the cluster and the mutant phenotyping results provide some clues. First, the *GALLAC* cluster seems to have important regulatory functions, which can help finetune metabolic fluxes and growth. We demonstrate that the cluster-encoded transcription factor Lac9_2 is important for onset of galactose and lactose growth, as deletion of *LAC9_2* leads to increased lag phases on both carbon sources. However, as *lac9_2Δ* cells eventually grow, Lac9_2 cannot be solely responsible for expression of the metabolic genes. Moreover, Lac9 binding motifs were only found in the promoters of *GALLAC* genes, suggesting that other transcriptional activators are responsible for induction of the *GAL* and *LAC* cluster genes.

**Fig 7:**
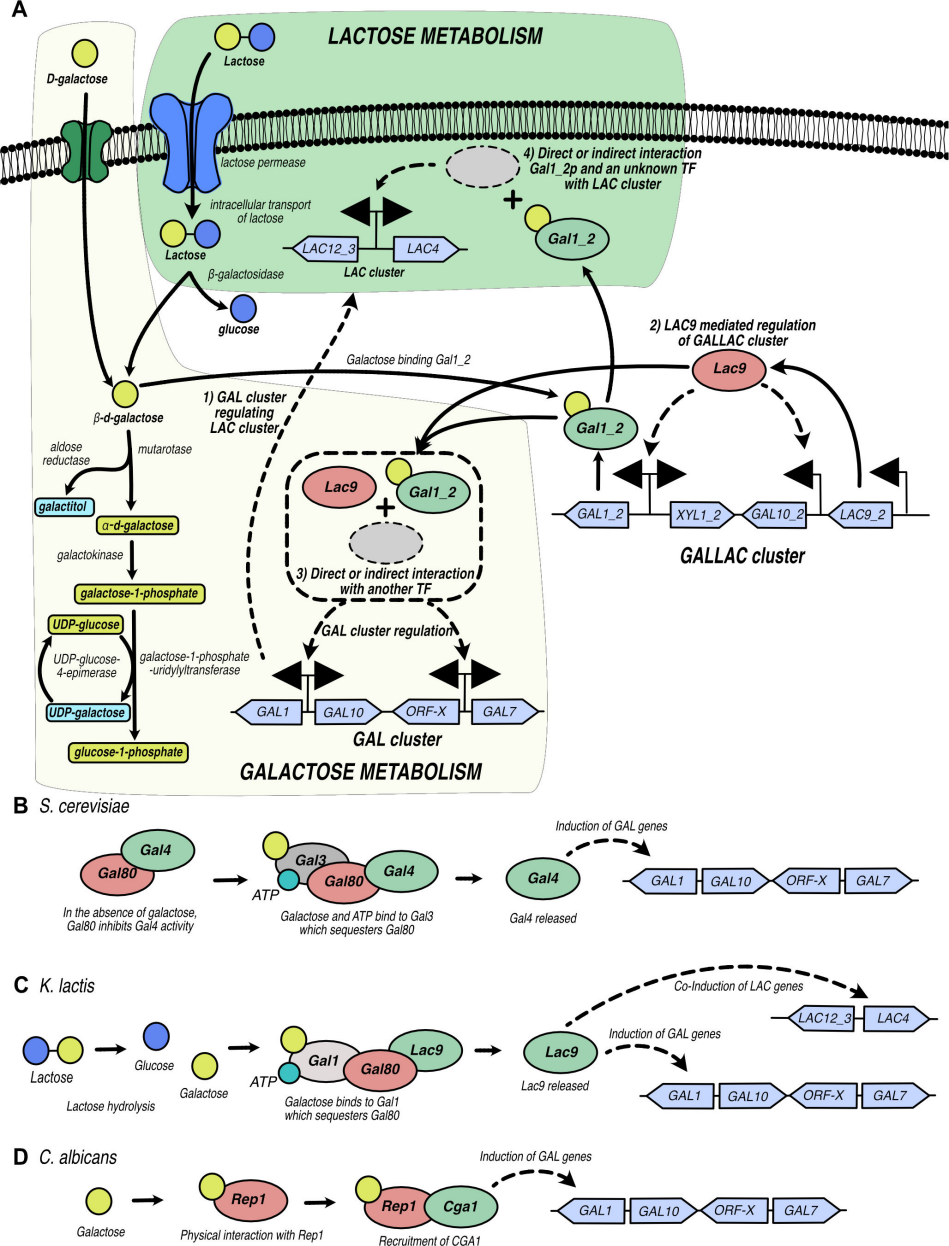
Graphic representation of regulatory mechanisms in *C. intermedia* and other yeast species. (A) Depiction of lactose (green box) and galactose (light yellow box) metabolism in *C. intermedia*. Instances of cluster interdependence are depicted with 1) the *GAL* cluster having a regulatory effect on the *LAC* cluster as *galΔ* cannot grow on lactose; 2) regulation of *GALLAC* cluster by the transcription factor *Ci*Lac9; 3) on galactose, Lac9 and Gal1_2 interact directly or indirectly resulting in the regulation of *GAL* cluster gene(s), thus affecting *C. intermedia*’s growth; and 4) on lactose, Gal1_2 from the *GALLAC* cluster regulates the *LAC* cluster at a transcriptional level. This effect of Gal1_2 can be speculated to be indirect due to the inability of Gal1_2 to bind DNA or protein based on predicted structure. Graphical representation also illustrates the overflow metabolism in *C. intermedia* through aldose reductase-mediated conversion of galactose to galactitol. (B) Regulation of galactose metabolism in *S. cerevisiae* by the Gal3-Gal80-Gal4 system where galactose and ATP induce Gal3 to bind Gal80 resulting in the activation of Gal4. Thus, Gal4 induces expression of the structural *GAL* genes. (C) Regulation of galactose and lactose genes in *K. lactis* mediated by the bi-functional Gal1. The *Sc*Gal3 homolog in *K. lactis* (Gal1) is induced by galactose (or galactose derived from lactose) resulting in sequestering Gal80 and relieving Lac9, which in turn activates the interconnected galactose and lactose metabolic genes in this yeast. (D) Graphic representation of the *C. albicans* galactose regulatory system through Rep1 and Cga1. Galactose physically binds to Rep1 resulting in recruitment of Cga1, and the complex ultimately induces the structural genes responsible for galactose metabolism in this yeast.

In addition to Lac9_2, Gal1_2 from the *GALLAC* cluster seems to be an important regulator of galactose and lactose growth. The bioinformatic analysis strongly suggests that *GAL1_2* in *C. intermedia* formed through gene duplication and divergence from the *GAL1* gene in the *GAL* cluster. Our results also show that Gal1_2 is essential for *LAC4* transcription and, in extension, lactase activity and lactose growth, whereas deletion of *GAL1_2* alone did not abolish *GAL1* expression and galactose growth. Although we have not provided evidence for a direct regulatory role of Gal1_2, these results suggest that the original Gal1 has maintained the function as the main galactokinase, while Gal1_2 has taken on the role as an important regulator. This evolutionary trajectory mirrors the path taken by Gal1 and Gal3 in *S. cerevisiae* (37), but with a crucial distinction: the Gal1 proteins in *C. intermedia* have evolved in response to both lactose and galactose. On galactose, impairing growth required the deletion of both *GAL1_2* and *LAC9_2*, whereas the deletion of *GAL1_2* alone was sufficient to impair growth on lactose. This suggests that the yeast senses and regulates the expression of genes for galactose and lactose metabolism somewhat differently. Since Gal1_2 does not have a DNA binding capacity, we hypothesize that Gal1_2 binds galactose and thereafter activates unknown transcription factor(s) that ultimately bind and induce expression from the *LAC* and *GAL* clusters (Fig. 7). It should be noted that the *GAL* cluster likely also has a regulatory role, as indicated by the fact that the *galΔ* strain grows poorly on lactose, even though it possesses an intact *LAC* cluster and a functional glycolysis pathway for glucose catabolism. Although many details are still to be elucidated, it is clear that *C. intermedia* has developed a way of regulating its galactose and lactose metabolism that differs from other yeast species studied to date, including the Gal3-Gal80-Gal4 regulon in *S. cerevisiae* (40), the Gal1-Gal80-Lac9 equivalent in *K. lactis* (31), and the Rep1-Cga1 regulatory complex in *C. albicans* (20) (Fig. 7). Future research will include identifying these unknown transcription factors and fully elucidating the roles of Lac9_2 and Gal1_2 in sensing, signaling, and regulating the cellular response to changes in the nutritional environment.

Another interesting feature of the *GALLAC* cluster is the *XYL1_2* gene encoding an aldose reductase. Although no galactitol or other intermediates of an oxidoreductive pathway were detected in the WT under the growth conditions assessed, several of the constructed mutants (in particular, *galΔ* and *gal1Δ*) accumulate galactitol upon growth on lactose. In *S. cerevisiae*, galactitol functions as an overflow metabolite ensuring that cells avoid accumulation of galactose-1-phosphate, a known toxic intermediate of the Leloir pathway in the cell (13, 41), and it is reasonable to assume that the same is true for *C. intermedia*. Moreover, it is interesting to note that aldose reductases can directly convert *β*-D-galactose, the hydrolysis product of lactose, whereas galactokinase requires *β*-D-galactose conversion into *α*-D-galactose before it can be metabolized via the Leloir pathway. We speculate that induction of an aldose reductase gene in tandem with the *LAC* and *GAL* genes in response to lactose (and galactose) can be an efficient way to quickly metabolize these sugars, providing a growth advantage in competitive lactose-rich environments.

In addition to the basic scientific questions that can be answered by studying evolution and sugar metabolism in lactose-growing yeast species, these yeasts can also be used as cell factories in industrial biotechnology processes. Here, a better understanding of the underlying genetics for this trait enables metabolic engineering to optimize the conversion of lactose-rich whey into value-added products. The dairy yeasts *K. lactis* and *K. marxianus* have been developed and used for whey-based production of ethanol (42), recombinant proteins (43), and bulk chemicals such as ethyl acetate (44), while exploration of new lactose-metabolizing yeasts allows for additional product diversification. With lactose as substrate, a carbon-partition strategy can be used for bioproduction, where the glucose moiety is converted into energy and yeast biomass and the galactose moiety is steered into production of the wanted metabolite, or vice versa (45). Through this strategy, the non-conventional yeast *C. intermedia* can also be explored to produce various growth-coupled metabolites, including galactitol and derivatives thereof.

In conclusion, our work on the non-conventional, lactose-metabolizing yeast *C. intermedia* has paved the way toward a better understanding of the galactose and lactose metabolism in this relatively understudied species. To the best of our knowledge, we show for the first time that gene duplication and divergence resulted in the formation of a unique *GALLAC* cluster and its essential role in galactose and lactose metabolism in this yeast, providing new insights of how organisms can evolve metabolic pathways and regulatory networks. In addition, the proven ability of *C. intermedia* to grow relatively well on lactose establishes this yeast as an interesting lactose-assimilating species also for future industrial applications.

## MATERIALS AND METHODS

### Culture conditions and molecular techniques

For amplification of plasmids, *E. coli* was grown on LB medium (1% tryptone, 1% NaCl, and 0.5% yeast extract) containing ampicillin (100 µg/mL) for plasmid selection.

*C. intermedia* CBS 141442 was grown in YPD medium (1% yeast extract, 2% bactopeptone, and 2% glucose) prior to yeast transformation using the split marker technique as described previously (30). Using this technique, deletion cassettes were constructed as two partially overlapping fragments, each containing half of the selection marker fused to either upstream or downstream sequences of the target gene. Deletion fragments were transformed using electroporation (Bio-Rad Micropulse electroporator) (30). After transformation, cells were plated on YPD agar containing 200 µg/mL nourseothricin to select for integration and expression of the *CaNAT1* selection marker.

Colony PCR was used to identify transformants with correct gene deletions, where single colonies were resuspended in 50 µL dH_2_O using a sterile toothpick and then heated to 90°C for 10 min. After cooling to 12°C, 2 µL of each suspension was used as a template for PCR using PHIRE II polymerase (Thermo Fisher Scientific, USA). For each mutant, three PCR primers were used, where the first primer was designed to hybridize to the genome outside the flanking region, the second to the marker gene and the third to the targeted gene (negative control). Since replicative plasmids and overexpression cassettes are missing for *C. intermedia*, we have not been able to perform complementation analyses to ensure that the targeted deletions are linked to the phenotypes we observe. Instead, three individual transformants for each deleted gene have been carefully tested, to ensure that they exhibit the same growth phenotype. To construct the double gene deletion mutant (*lac9_2*, the split marker method was used twice in the same strain background, first employing the split *CaNAT1* selection marker as described above and then a split KanMX selection marker PCR amplified from the plasmid pTO149_RFP_CauNEO developed for *Candida auris*) (46). Correctly assembled and genome-integrated KanMX markers gave rise to *C. intermedia* transformants resistant to the antibiotic Geneticin (200 µg/mL).

For complementation tests in *S. cerevisiae*, *C. intermedia GAL1* and *GAL1_2* genes were synthesized and cloned in a vector backbone (pESC-URA; GenScript Biotech, New Jersey, USA). Codon CTG were adjusted to alternate codon prior to optimization of the complete gene for expression in *S. cerevisiae* using the GenSmart Codon Optimization tool (GenScript Biotech, New Jersey, USA). *S. cerevisiae* BY4741 *GAL1* knockouts used for complementation experiment were grown on YP media with 2% glucose and transformed with above-mentioned plasmids using LiAc/PEG heat-shock method (47). Transformants were selected on agar plates with YNB -uracil and 2% glucose, restreaked, and then tested for growth in liquid YNB-URA media with 2% galactose in GrowthProfiler at 30°C and 250 rpm. *S. cerevisiae* BY4741 *gal1Δ* transformed with p426 (empty vector with *URA3* as selection marker) was used as negative control.

### Growth experiments

#### Growth profiler

To follow growth over time for *C. intermedia* CBS 141442 and the other yeasts characterized in this work, strains were precultured at 30°C, 180 rpm overnight in synthetic defined minimal Verduyn media (48) containing 2% glucose (wt/vol). Precultured cells were then inoculated in 250 µL minimal media supplemented with 20 g/L carbon source to a starting OD_600_  =  0.1. All yeast strains were grown in biological triplicates in a 96-well plate setup in a GrowthProfiler 960 (Enzyscreen, Netherlands). “Green Values” (GV) measured by the GrowthProfiler correspond to growth based on pixel counts, and GV changes were recorded every 30 min for the duration of the experiment and at 30°C and 150 rpm.

#### Cell growth quantifier (CGQ)

Growth characterization was also performed in shake flasks using CGQ (Scientific Bioprocessing, Germany) (49). Wild-type and mutant strains were precultured at 30°C, 200 rpm overnight in synthetic defined minimal Verduyn media containing 2% glucose (wt/vol), followed by inoculation of 25 mL of minimal medium supplemented with 2% carbon source in 100-mL shake flasks to a starting OD_600_ = 0.1. Growth was quantified as “Scatter values” by the CGQ system (49). Scatter values were recorded for 10 days at 30°C and 200 rpm for each strain growth in biological triplicates, and sampling was performed for sugar and polyol analyses.

### Lactase activity assay

β-Galactosidase activity was determined using the Yeast β-Galactosidase Assay Kit (Thermo Fisher Scientific, USA) following the manufacturer’s instructions. Cells were harvested at different timepoints during growth and tested for lactase activity. A working solution was prepared by mixing equal amounts of 2× β-Galactosidase Assay Buffer [containing ortho-nitrophenyl-β-galactoside (ONPG)] and Yeast Protein Extraction Reagent. The reaction was initiated by mixing 100 μL of working solution with 100 μL cell culture and incubated for 30 min at 37°C in a thermomixer. After 30 min, cell mix was centrifuged at 5,000 rpm for 3 min and the supernatant was analyzed for lactase activity by measuring o-nitrophenol release from ONPG at 420 nm in a microplate reader (FLU-Ostar Omega-BMG LabTech, Ortenberg, Germany).

### Determination of sugar and polyol concentrations

Sugar and galactitol concentrations were measured using a Dionex high-performance liquid chromatography system equipped with an RID-10A Refractive Index Detector and an Aminex HPX-87H Carbohydrate Analysis Column (Bio-Rad Laboratories). Analysis was performed with the column at 80°C and 5 mM H_2_SO_4_ as mobile phase at a constant flow rate of 0.8 mL/min. Culture samples were pelleted prior to analysis, following which the supernatant was passed through a 0.22-µm polyether sulfone syringe filter. Chromatogram peaks were identified and integrated using the Chromeleon v6.8 (Dionex) software and quantified against prepared analytical standards.

### Comparative genomics and evolutionary mapping

We established the blast database for 332 yeast species based on the work of Shen et al. (1). Then, we used tblastn [with threshold pident (percentage of identical matches) > 40% and qcovs (Query Coverage Per Subject) > 60%] to get gene hits for each specific gene in three clusters against 332 yeast species. Based on the generated data, we further mapped gene hits from species to clade levels.

To investigate the evolution of genes in the *GALLAC* cluster, a comprehensive pipeline based on the work of Goncalves and colleagues was developed (50). For each candidate gene in the *GALLAC* cluster, BLASTP was run against the NCBI non-redundant (nr) protein sequence database and homologs were selected according to the top 300 BLAST hits to each query sequence. These homologs were aligned with MAFFT v7.310 (51) using default settings for multiple sequence alignment. Poorly aligned regions were removed with trimAl (52) using the “-automated1” option. Subsequently, phylogenetic trees were built using IQ-TREE v1.6.12 (53) with 1,000 ultrafast bootstrapping replicates (54). Each tree was rooted at the midpoint using a customized script combining R packages ape v5.4-1 and phangorn v2.5.5. Finally, the resulting phylogenies were visualized using iTol v5 (55).

### Transcription factor binding motif analysis

To determine the binding motifs of transcription factors in promoter regions of the *GAL*, *LAC*, and *GALLAC* cluster, MEME (version 5.5.43) promoter binding motif analysis was used. Promoter regions of all genes from the three clusters were added as query sequences with the following constraints: maximum number of motifs = 5, maximum length of motif = 25 bases, any number of motif repetitions (-anr), and background model = 0—order model of sequences. Motif(s) derived from this analysis were then fed as input to Tomtom (56) (version 5.5.4) to compare with Yeastract (57) database.

### RNA sequencing

Transcriptomics using RNA sequencing was performed as previously described (28). In brief, *C. intermedia* CBS 141442 was grown in controlled stirred 1-L bioreactor vessels (DASGIP, Eppendorf, Hamburg, Germany) containing 500 mL synthetic defined minimal Verduyn media with 2% glucose, galactose, or lactose. Reactor conditions were maintained as follows: Temp = 30°C; pH = 5.5 (maintained with 2M Potassium Hydroxide); Aeration = 1 Vessel Volume per Minute; stirring = 300 rpm.

#### RNA extraction

For RNA extraction, samples (10 mL) were collected when the dissolved oxygen of the culture was 35%–40% (vol/vol). After washing the cells, the pellets were immediately frozen using liquid nitrogen. Frozen pellets were stored at −80°C until extraction. The frozen pellets were thawed in 500 µL of TRIzol (Ambion—Foster City, CA, USA) and thoroughly resuspended. Then, cells were lysed in 2-mL tubes with Lysing Matrix C (MP Biomedical, Santa Ana, CA, USA) in a FastPrep FP120 (Savant, Carlsbad, CA, USA) for five cycles, at intensity 5.5 for 30 s. Tubes were cooled on ice for a minute between cycles and resuspended once again in 500 µL of TRIzol and vortexed thoroughly. After incubation at room temperature for 5 min, tubes were centrifuged for 10 min at 12,000 rpm and 4°C. Chloroform was added to the collected supernatants (200 µL of chloroform per mL of supernatant) and vortexed vigorously for 30 s. After centrifugation for 15 min at 12,000 rpm, 4°C, the top clear aqueous phase was collected and transferred to a new RNase-free tube, to which an equal amount of absolute ethanol was slowly added while mixing. Each sample was loaded into an RNeasy column (RNeasy Mini Kit, Qiagen—Hilden, Germany), and further steps followed the protocol of the manufacturer. The RNA was eluted with RNase-free water, and samples were stored at −80°C until use.

#### Data analysis

RNA samples were analyzed in a TapeStation (Agilent, Santa Clara, CA, USA), and only samples with RNA integrity number above 8 were used for library preparation. Sequencing using the HiSeq 2500 system (Illumina Inc.—San Diego, CA, USA), with paired-end 125-bp read length, and v4 sequencing chemistry was followed by quality control of read data using the software FastQC version 0.11.5 (58). Software Star version 2.5.2b (59) was used to map reads to the reference genome. Gene counts were normalized with weighted trimmed mean of *M* values using the calcNormFactor function from the package edgeR (60), and Limma package (61) was used to transform and make data suitable for linear modeling. The estimated *P* values were corrected for multiple testing with the Benjamini-Hochberg procedure, and genes were considered significant if the adjusted *P* values were lower than 0.05. The raw counts were filtered such that genes with CPM > 3.84 in at least 12% (5/43) of the samples were retained. The R function “varianceStabilizingTransformation()” from R package “DESeq2” (62) was used to convert raw counts to variance-stabilized counts. Expression data for *C. intermedia* on galactose and lactose were normalized using glucose as control condition.

### Gene expression analysis using qPCR

Primers used for mRNA quantification using qPCR are listed in Table S4. Primers were designed using Primer3 (https://primer3.ut.ee/) and were checked for efficiency. Only primers having efficiency rates between 90% and 110% were used for qPCR. Cultures with a starting OD_600_ = 1 were grown at 30°C and 200 rpm in 100-mL shake flasks containing 25–100 mL synthetic defined minimal Verduyn media containing either 2% glucose (control), galactose, or lactose as carbon source. Cells were harvested for each strain at lag, early log, and late log phases, taking three biological replicates. Harvested cells were pelleted by centrifugation at 4°C for 5 min at 5,000 rpm and washed twice by resuspending in ice-cold sterile dH_2_O water and centrifugation. Cell pellet was snap frozen using liquid nitrogen and stored at −80°C for cDNA synthesis. RNA extraction was performed as described for RNA sequencing above. cDNA synthesis and real-time qPCR analysis were performed using a Maxima H Minus First Strand cDNA Synthesis Kit (Thermo Fisher) and Maxima SYBR Green/Fluorescein qPCR Master Mix (2×) (Thermo Fisher), according to the manufacturer’s instruction. Fold change was calculated using the delta-delta Ct method (2^-^*^ΔΔ^*^Ct^) with expression values in glucose as control condition and Ci*ACT1* as the reference gene for normalization.

### Statistical analysis

Statistical analysis for qPCR data were performed using R software, function t.test for two-tailed *t*-test. Statistical significance was established at *P* < 0.05 and marked by *P < 0.05, **P < 0.01, and ***P < 0.001.

## Data Availability

The RNA-Seq data sets are available in the ENA with the accession number E-MTAB-6670.

## SUPPLEMENTAL MATERIAL

Supplemental figures aem.01135-24-s0001.docx Figures S1 to S6.

Supplemental tables aem.01135-24-s0002.xlsx Tables S1 to S4.

ASM does not own the copyrights to Supplemental Material that may be linked to, or accessed through, an article. The authors have granted ASM a non-exclusive, world-wide license to publish the Supplemental Material files. Please contact the corresponding author directly for reuse.

## References

[1] Shen X-X, Opulente DA, Kominek J, Zhou X, Steenwyk JL, Buh KV, Haase MAB, Wisecaver JH, Wang M, Doering DT, et al.. 2018. Tempo and mode of genome evolution in the budding yeast subphylum. Cell 175:1533–1545. doi:10.1016/j.cell.2018.10.023 30415838 PMC6291210

[2] Schaffrath R, Breunig KD. 2000. Genetics and molecular physiology of the yeast Kluyveromyces lactis. Fungal Genet Biol 30:173–190. doi:10.1006/fgbi.2000.1221 11035939

[3] Gödecke A, Zachariae W, Arvanitidis A, Breunig KD. 1991. Coregulation of the Kluyveromyces lactis lactose permease and beta-galactosidase genes is achieved by interaction of multiple LAC9 binding sites in a 2.6 kbp divergent promoter. Nucleic Acids Res 19:5351–5358. doi:10.1093/nar/19.19.5351 1923819 PMC328898

[4] Lane MM, Burke N, Karreman R, Wolfe KH, O’Byrne CP, Morrissey JP. 2011. Physiological and metabolic diversity in the yeast Kluyveromyces marxianus. Antonie Van Leeuwenhoek 100:507–519. doi:10.1007/s10482-011-9606-x 21674230

[5] Varela JA, Puricelli M, Ortiz-Merino RA, Giacomobono R, Braun-Galleani S, Wolfe KH, Morrissey JP. 2019. Origin of lactose fermentation in Kluyveromyces lactis by interspecies transfer of a neo-functionalized gene cluster during domestication. Curr Biol 29:4284–4290. doi:10.1016/j.cub.2019.10.044 31813610 PMC6926475

[6] Marcus JF, DeMarsh TA, Alcaine SD. 2021. Upcycling of whey permeate through yeast- and mold-driven fermentations under anoxic and oxic conditions. Ferment 7:16. doi:10.3390/fermentation7010016

[7] Thoden JB, Holden HM. 2007. The molecular architecture of glucose-1-phosphate uridylyltransferase. Protein Sci 16:432–440. doi:10.1110/ps.062626007 17322528 PMC2203317

[8] Slot JC, Rokas A. 2010. Multiple GAL pathway gene clusters evolved independently and by different mechanisms in fungi. Proc Natl Acad Sci U S A 107:10136–10141. doi:10.1073/pnas.0914418107 20479238 PMC2890473

[9] Mojzita D, Herold S, Metz B, Seiboth B, Richard P. 2012. L-xylo-3-hexulose reductase is the missing link in the oxidoreductive pathway for D-galactose catabolism in filamentous fungi. J Biol Chem 287:26010–26018. doi:10.1074/jbc.M112.372755 22654107 PMC3406684

[10] Gruben BS, Zhou M, de Vries RP. 2012. GalX regulates the D-galactose oxido-reductive pathway in aspergillus niger. FEBS Lett 586:3980–3985. doi:10.1016/j.febslet.2012.09.029 23063944

[11] Harrison MC, LaBella AL, Hittinger CT, Rokas A. 2022. The evolution of the GALactose utilization pathway in budding yeasts. Trends Genet 38:97–106. doi:10.1016/j.tig.2021.08.013 34538504 PMC8678326

[12] Rokas A, Wisecaver JH, Lind AL. 2018. The birth, evolution and death of metabolic gene clusters in fungi. Nat Rev Microbiol 16:731–744. doi:10.1038/s41579-018-0075-3 30194403

[13] Jongh WA, Bro C, Ostergaard S, Regenberg B, Olsson L, Nielsen J. 2008. The roles of galactitol, galactose-1-phosphate, and phosphoglucomutase in galactose-induced toxicity in Saccharomyces cerevisiae. Biotechnol Bioeng 101:317–326. doi:10.1002/bit.21890 18421797

[14] Martchenko M, Levitin A, Hogues H, Nantel A, Whiteway M. 2007. Transcriptional rewiring of fungal galactose-metabolism circuitry. Curr Biol 17:1007–1013. doi:10.1016/j.cub.2007.05.017 17540568 PMC3842258

[15] Van Ende M, Wijnants S, Van Dijck P. 2019. Sugar sensing and signaling in Candida albicans and Candida glabrata. Front Microbiol 10:99. doi:10.3389/fmicb.2019.00099 30761119 PMC6363656

[16] Peng G, Hopper JE. 2002. Gene activation by interaction of an inhibitor with a cytoplasmic signaling protein. Proc Natl Acad Sci USA 99:8548–8553. doi:10.1073/pnas.142100099 12084916 PMC124307

[17] Bhat PJ, Murthy TV. 2001. Transcriptional control of the GAL/MEL regulon of yeast Saccharomyces cerevisiae: mechanism of galactose-mediated signal transduction. Mol Microbiol 40:1059–1066. doi:10.1046/j.1365-2958.2001.02421.x 11401712

[18] Meyer J, Walker-Jonah A, Hollenberg CP. 1991. Galactokinase encoded by GAL1 is a bifunctional protein required for induction of the GAL genes in Kluyveromyces lactis and is able to suppress the gal3 phenotype in Saccharomyces cerevisiae. Mol Cell Biol 11:5454–5461. doi:10.1128/mcb.11.11.5454-5461.1991 1922058 PMC361914

[19] Dalal CK, Zuleta IA, Mitchell KF, Andes DR, El-Samad H, Johnson AD. 2016. Transcriptional rewiring over evolutionary timescales changes quantitative and qualitative properties of gene expression. Elife 5:e18981. doi:10.7554/eLife.18981 27614020 PMC5067116

[20] Sun X, Yu J, Zhu C, Mo X, Sun Q, Yang D, Su C, Lu Y. 2023. Recognition of galactose by a scaffold protein recruits a transcriptional activator for the GAL regulon induction in Candida albicans Elife 12:e84155. doi:10.7554/eLife.84155 36723430 PMC9925049

[21] Wu J, Hu J, Zhao S, He M, Hu G, Ge X, Peng N. 2018. Single-cell protein and xylitol production by a novel yeast strain Candida intermedia FL023 from lignocellulosic hydrolysates and xylose. Appl Biochem Biotechnol 185:163–178. doi:10.1007/s12010-017-2644-8 29098561 PMC5937888

[22] Gárdonyi M, Osterberg M, Rodrigues C, Spencer-Martins I, Hahn-Hägerdal B. 2003. High capacity xylose transport in Candida intermedia PYCC 4715. FEMS Yeast Res 3:45–52. doi:10.1111/j.1567-1364.2003.tb00137.x 12702245

[23] Fonseca C, Olofsson K, Ferreira C, Runquist D, Fonseca LL, Hahn-Hägerdal B, Lidén G. 2011. The glucose/xylose facilitator Gxf1 from Candida intermedia expressed in a xylose-fermenting industrial strain of Saccharomyces cerevisiae increases xylose uptake in SSCF of wheat straw. Enzyme Microb Technol 48:518–525. doi:10.1016/j.enzmictec.2011.02.010 22113025

[24] Moreno AD, Carbone A, Pavone R, Olsson L, Geijer C. 2019. Evolutionary engineered Candida intermedia exhibits improved xylose utilization and robustness to lignocellulose-derived inhibitors and ethanol. Appl Microbiol Biotechnol 103:1405–1416. doi:10.1007/s00253-018-9528-x 30498977 PMC6394480

[25] Mayr P, Brüggler K, Kulbe KD, Nidetzky B. 2000. D-Xylose metabolism by Candida intermedia: isolation and characterisation of two forms of aldose reductase with different coenzyme specificities. J Chromatogr B Biomed Sci Appl 737:195–202. doi:10.1016/s0378-4347(99)00380-1 10681056

[26] Nidetzky B, Brüggler K, Kratzer R, Mayr P. 2003. Multiple forms of xylose reductase in Candida intermedia: comparison of their functional properties using quantitative structure-activity relationships, steady-state kinetic analysis, and pH studies. J Agric Food Chem 51:7930–7935. doi:10.1021/jf034426j 14690376

[27] Yönten V, Aktaş N. 2014. Exploring the optimum conditions for maximizing the microbial growth of Candida intermedia by response surface methodology. Prep Biochem Biotechnol 44:26–39. doi:10.1080/10826068.2013.782044 24117150

[28] Geijer C, Faria-Oliveira F, Moreno AD, Stenberg S, Mazurkewich S, Olsson L. 2020. Genomic and transcriptomic analysis of Candida intermedia reveals the genetic determinants for its xylose-converting capacity. Biotechnol Biofuels 13:48. doi:10.1186/s13068-020-1663-9 32190113 PMC7068945

[29] Moreno AD, Tellgren-Roth C, Soler L, Dainat J, Olsson L, Geijer C. 2017. Complete genome sequences of the xylose-fermenting Candida intermedia strains CBS 141442 and PYCC 4715. Genome Announc 5:e00138-17. doi:10.1128/genomeA.00138-17 28385851 PMC5383899

[30] Peri KVR, Faria-Oliveira F, Larsson A, Plovie A, Papon N, Geijer C. 2023. Split-marker-mediated genome editing improves homologous recombination frequency in the CTG clade yeast Candida intermedia. FEMS Yeast Res 23:foad016. doi:10.1093/femsyr/foad016 36893808 PMC10035504

[31] Wray LV, Witte MM, Dickson RC, Riley MI. 1987. Characterization of a positive regulatory gene, LAC9, that controls induction of the lactose-galactose regulon of Kluyveromyces lactis: structural and functional relationships to GAL4 of Saccharomyces cerevisiae. Mol Cell Biol 7:1111–1121. doi:10.1128/mcb.7.3.1111-1121.1987 3550430 PMC365183

[32] Douglas HC, Hawthorne DC. 1964. Enzymatic expression and genetic linkage of genes controlling galactose utilization in Saccharomyces. Genetics 49:837–844. doi:10.1093/genetics/49.5.837 14158615 PMC1210618

[33] Bailey TL, Johnson J, Grant CE, Noble WS. 2015. The MEME suite. Nucleic Acids Res 43:W39–W49. doi:10.1093/nar/gkv416 25953851 PMC4489269

[34] Nehlin JO, Carlberg M, Ronne H. 1991. Control of yeast GAL genes by MIG1 repressor: a transcriptional cascade in the glucose response. EMBO J 10:3373–3377. doi:10.1002/j.1460-2075.1991.tb04901.x 1915298 PMC453065

[35] Thoden JB, Sellick CA, Timson DJ, Reece RJ, Holden HM. 2005. Molecular structure of Saccharomyces cerevisiae Gal1p, a bifunctional galactokinase and transcriptional inducer. J Biol Chem 280:36905–36911. doi:10.1074/jbc.M508446200 16115868

[36] Jumper J, Evans R, Pritzel A, Green T, Figurnov M, Ronneberger O, Tunyasuvunakool K, Bates R, Žídek A, Potapenko A, et al.. 2021. Highly accurate protein structure prediction with AlphaFold. Nature New Biol 596:583–589. doi:10.1038/s41586-021-03819-2 PMC8371605 34265844

[37] Hittinger CT, Carroll SB. 2007. Gene duplication and the adaptive evolution of a classic genetic switch. Nature New Biol 449:677–681. doi:10.1038/nature06151 17928853

[38] Geronikou A, Larsen N, Lillevang SK, Jespersen L. 2022. Occurrence and identification of yeasts in production of white-brined cheese. Microorganisms 10:1079. doi:10.3390/microorganisms10061079 35744597 PMC9228510

[39] Tanji M, Namimatsu K, Kinoshita M, Motoshima H, Oda Y, Ohnishi M. 2004. Content and chemical compositions of cerebrosides in lactose-assimilating yeasts. Biosci Biotechnol Biochem 68:2205–2208. doi:10.1271/bbb.68.2205 15502372

[40] Yano K, Fukasawa T. 1997. Galactose-dependent reversible interaction of Gal3p with Gal80p in the induction pathway of Gal4p-activated genes of Saccharomyces cerevisiae. Proc Natl Acad Sci U S A 94:1721–1726. doi:10.1073/pnas.94.5.1721 9050845 PMC19983

[41] Jagtap SS, Bedekar AA, Liu JJ, Jin YS, Rao CV. 2019. Production of galactitol from galactose by the oleaginous yeast Rhodosporidium toruloides IFO0880. Biotechnol Biofuels 12:250. doi:10.1186/s13068-019-1586-5 31636709 PMC6798376

[42] Tesfaw A, Oner ET, Assefa F. 2021. Evaluating crude whey for bioethanol production using non-Saccharomyces yeast, Kluyveromyces marxianus. SN Appl Sci 3:42. doi:10.1007/s42452-020-03996-1

[43] Maullu C, Lampis G, Desogus A, Ingianni A, Rossolini GM, Pompei R. 1999. High-level production of heterologous protein by engineered yeasts grown in cottage cheese whey. Appl Environ Microbiol 65:2745–2747. doi:10.1128/AEM.65.6.2745-2747.1999 10347071 PMC91406

[44] Urit T, Stukert A, Bley T, Löser C. 2012. Formation of ethyl acetate by Kluyveromyces marxianus on whey during aerobic batch cultivation at specific trace element limitation. Appl Microbiol Biotechnol 96:1313–1323. doi:10.1007/s00253-012-4107-z 22573271

[45] Liu JJ, Zhang GC, Kwak S, Oh EJ, Yun EJ, Chomvong K, Cate JHD, Jin YS. 2019. Overcoming the thermodynamic equilibrium of an isomerization reaction through oxidoreductive reactions for biotransformation. Nat Commun 10:1356. doi:10.1038/s41467-019-09288-6 30902987 PMC6430769

[46] Santana DJ, O’Meara TR. 2021. Forward and reverse genetic dissection of morphogenesis identifies filament-competent Candida auris strains. Nat Commun 12:7197. doi:10.1038/s41467-021-27545-5 34893621 PMC8664941

[47] Gietz RD, Schiestl RH. 2007. High-efficiency yeast transformation using the LiAc/SS carrier DNA/PEG method. Nat Protoc 2:31–34. doi:10.1038/nprot.2007.13 17401334

[48] Verduyn C, Postma E, Scheffers WA, Van Dijken JP. 1992. Effect of benzoic acid on metabolic fluxes in yeasts: a continuous-culture study on the regulation of respiration and alcoholic fermentation. Yeast 8:501–517. doi:10.1002/yea.320080703 1523884

[49] Bruder S, Reifenrath M, Thomik T, Boles E, Herzog K. 2016. Parallelised online biomass monitoring in shake flasks enables efficient strain and carbon source dependent growth characterisation of Saccharomyces cerevisiae. Microb Cell Fact 15:127. doi:10.1186/s12934-016-0526-3 27455954 PMC4960845

[50] Gonçalves C, Wisecaver JH, Kominek J, Oom MS, Leandro MJ, Shen X-X, Opulente DA, Zhou X, Peris D, Kurtzman CP, Hittinger CT, Rokas A, Gonçalves P. 2018. Evidence for loss and reacquisition of alcoholic fermentation in a fructophilic yeast lineage. Elife 7. doi:10.7554/eLife.33034 PMC5897096 29648535

[51] Katoh K, Standley DM. 2013. MAFFT multiple sequence alignment software version 7: improvements in performance and usability. Mol Biol Evol 30:772–780. doi:10.1093/molbev/mst010 23329690 PMC3603318

[52] Capella-Gutiérrez S, Silla-Martínez JM, Gabaldón T. 2009. trimAl: a tool for automated alignment trimming in large-scale phylogenetic analyses. Bioinformatics 25:1972–1973. doi:10.1093/bioinformatics/btp348 19505945 PMC2712344

[53] Nguyen L-T, Schmidt HA, von Haeseler A, Minh BQ. 2015. IQ-TREE: a fast and effective stochastic algorithm for estimating maximum-likelihood phylogenies. Mol Biol Evol 32:268–274. doi:10.1093/molbev/msu300 25371430 PMC4271533

[54] Minh BQ, Nguyen MAT, von Haeseler A. 2013. Ultrafast approximation for phylogenetic bootstrap. Mol Biol Evol 30:1188–1195. doi:10.1093/molbev/mst024 23418397 PMC3670741

[55] Letunic I, Bork P. 2019. Interactive tree Of Life (iTOL) v4: recent updates and new developments. Nucleic Acids Res 47:W256–W259. doi:10.1093/nar/gkz239 30931475 PMC6602468

[56] Gupta S, Stamatoyannopoulos JA, Bailey TL, Noble WS. 2007. Quantifying similarity between motifs. Genome Biol 8:R24. doi:10.1186/gb-2007-8-2-r24 17324271 PMC1852410

[57] Teixeira MC, Viana R, Palma M, Oliveira J, Galocha M, Mota MN, Couceiro D, Pereira MG, Antunes M, Costa IV, Pais P, Parada C, Chaouiya C, Sá-Correia I, Monteiro PT. 2023. YEASTRACT+: a portal for the exploitation of global transcription regulation and metabolic model data in yeast biotechnology and pathogenesis. Nucleic Acids Res 51:D785–D791. doi:10.1093/nar/gkac1041 36350610 PMC9825512

[58] Andrews S. 2010. FastQC: a quality control tool for high throughput sequence data. Babraham Bioinformatics, Babraham Institute, Cambridge, United Kingdom

[59] Dobin A, Davis CA, Schlesinger F, Drenkow J, Zaleski C, Jha S, Batut P, Chaisson M, Gingeras TR. 2013. STAR: ultrafast universal RNA-seq aligner. Bioinformatics 29:15–21. doi:10.1093/bioinformatics/bts635 23104886 PMC3530905

[60] Robinson MD, McCarthy DJ, Smyth GK. 2010. edgeR: a bioconductor package for differential expression analysis of digital gene expression data. Bioinformatics 26:139–140. doi:10.1093/bioinformatics/btp616 19910308 PMC2796818

[61] Smyth GK. 2005. Limma: linear models for microarray data, p 397–420. In Bioinformatics and computational biology solutions using R and bioconductor. Springer.

[62] Love MI, Huber W, Anders S. 2014. Moderated estimation of fold change and dispersion for RNA-seq data with DESeq2. Genome Biol 15:550. doi:10.1186/s13059-014-0550-8 25516281 PMC4302049

