# Supplementary material for: A unique metabolic gene cluster regulates lactose and galactose metabolism in the yeast Candida intermedia
Source: Appl Environ Microbiol. 2024 Sep 6;90(10):e01135-24. doi: 10.1128/aem.01135-24 (PMC11497787; doi:10.1128/aem.01135-24)
Supplement: Supplemental figures — Figures S1 to S6. [file aem.01135-24-s0001.docx]

# Supplementary figures and figure legends


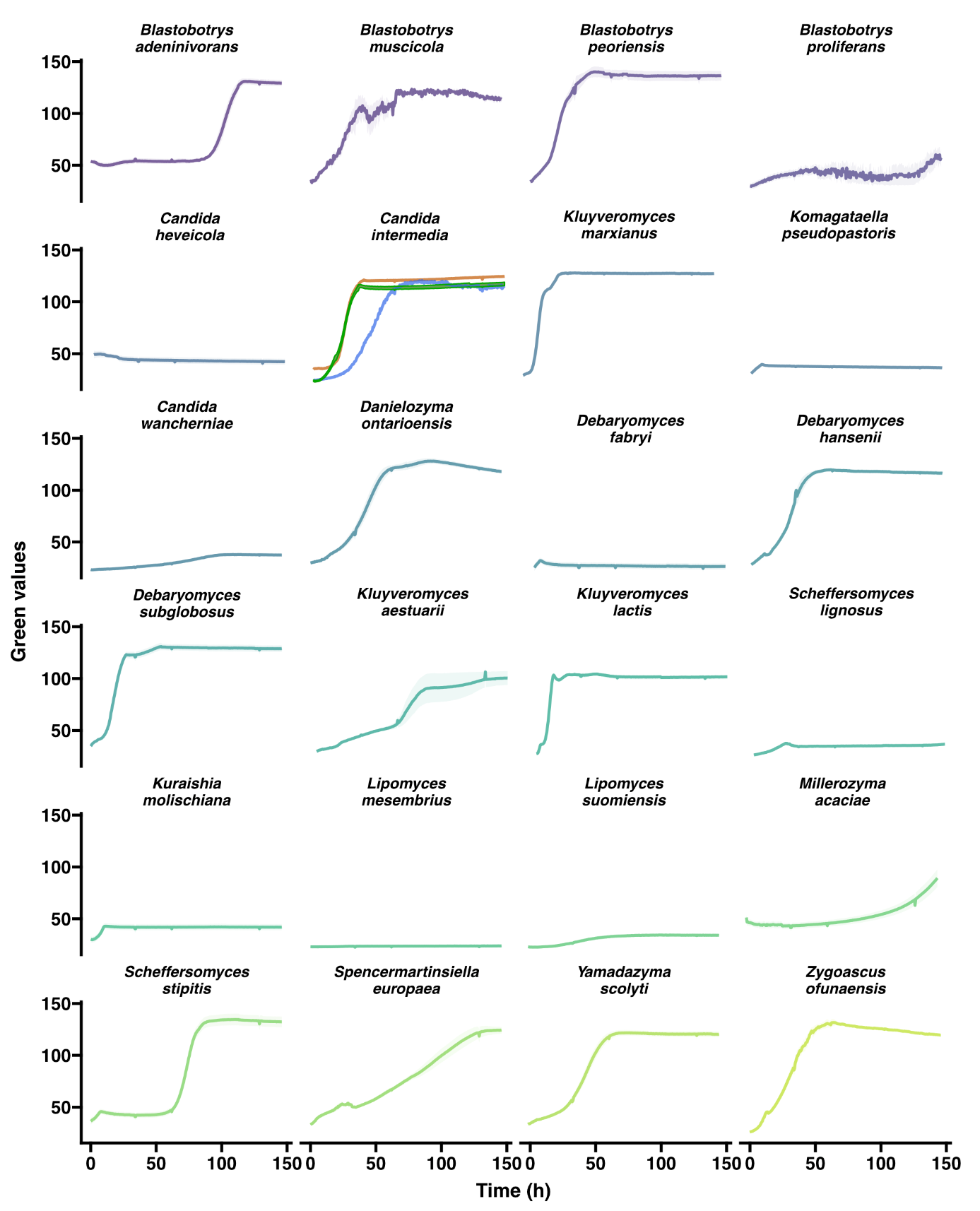


**Figure S1:** **Growth curves for 24 species identified as lactose-growing by Shen et al (1)**. The graphs depict data procured in GrowthProfiler in 96-well format, plotted as mean ± standard deviation (shaded region) for biological triplicates per strain. On y-axis final biomass yield is depicted in green values (G.V. - corresponding to growth based on pixel counts, as determined by a GrowthProfiler instrument) and is plotted against time (h) on x-axis. Three C. intermedia strains are shown in different colors (PYCC4715 in orange; CBS1442 in green; CBS572 in blue).


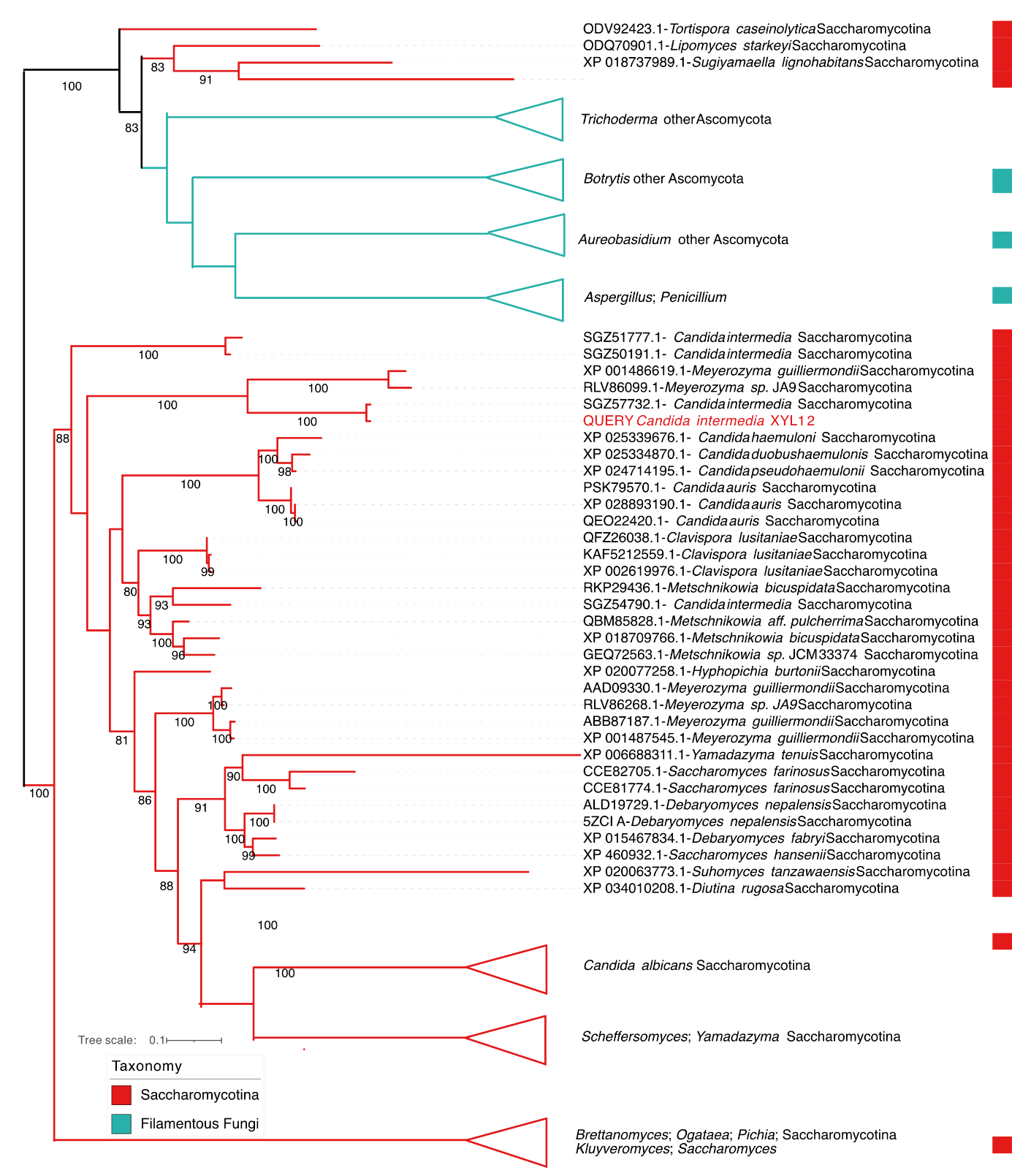


**Figure S2:** **Maximum likelihood phylogenetic tree depicting the origin and evolution of the XYL1_2 gene in C. intermedia.**


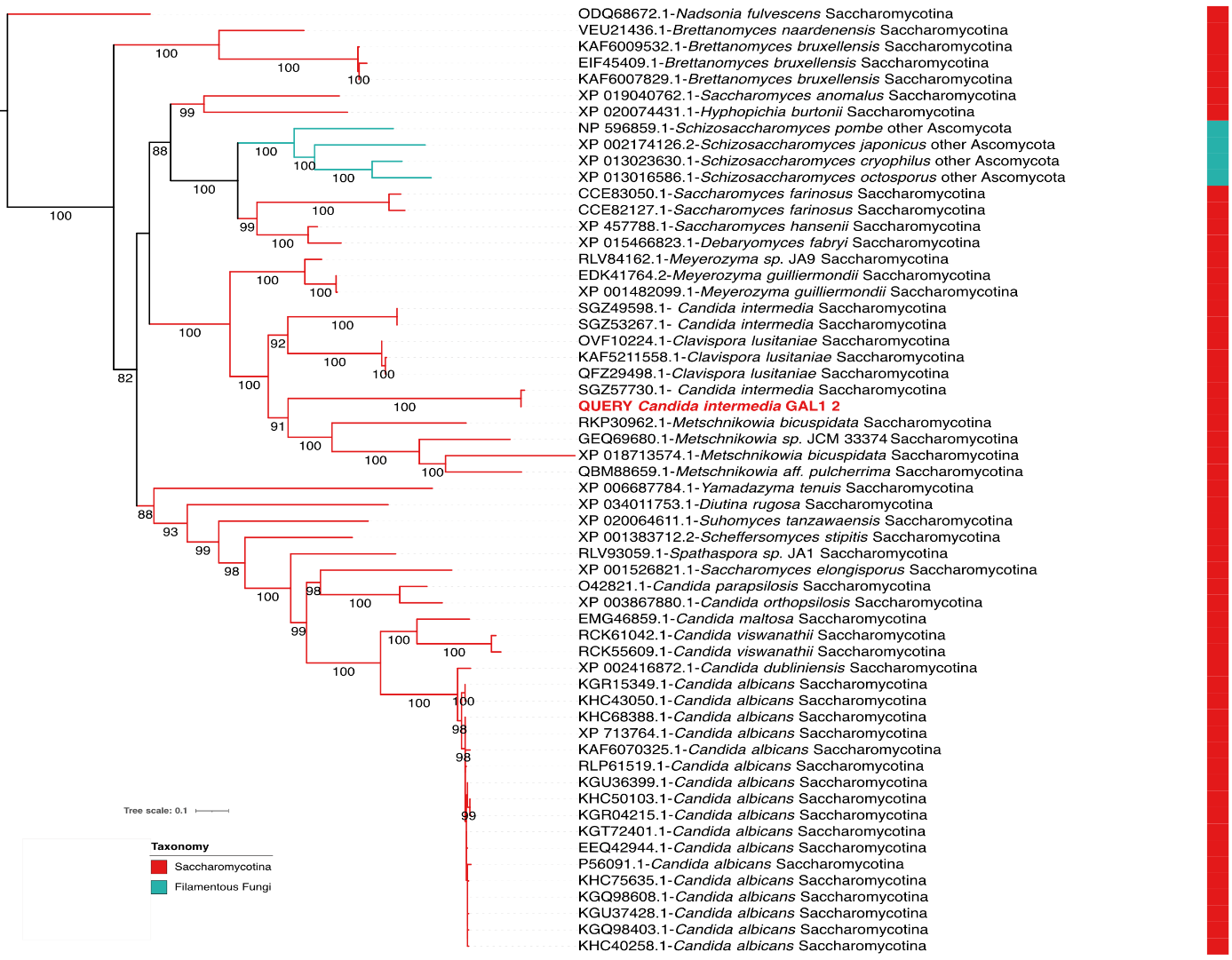


**Figure S3: Maximum Likelihood phylogenetic tree depicting origin and evolution of the GAL1_2 gene in C. intermedia.**


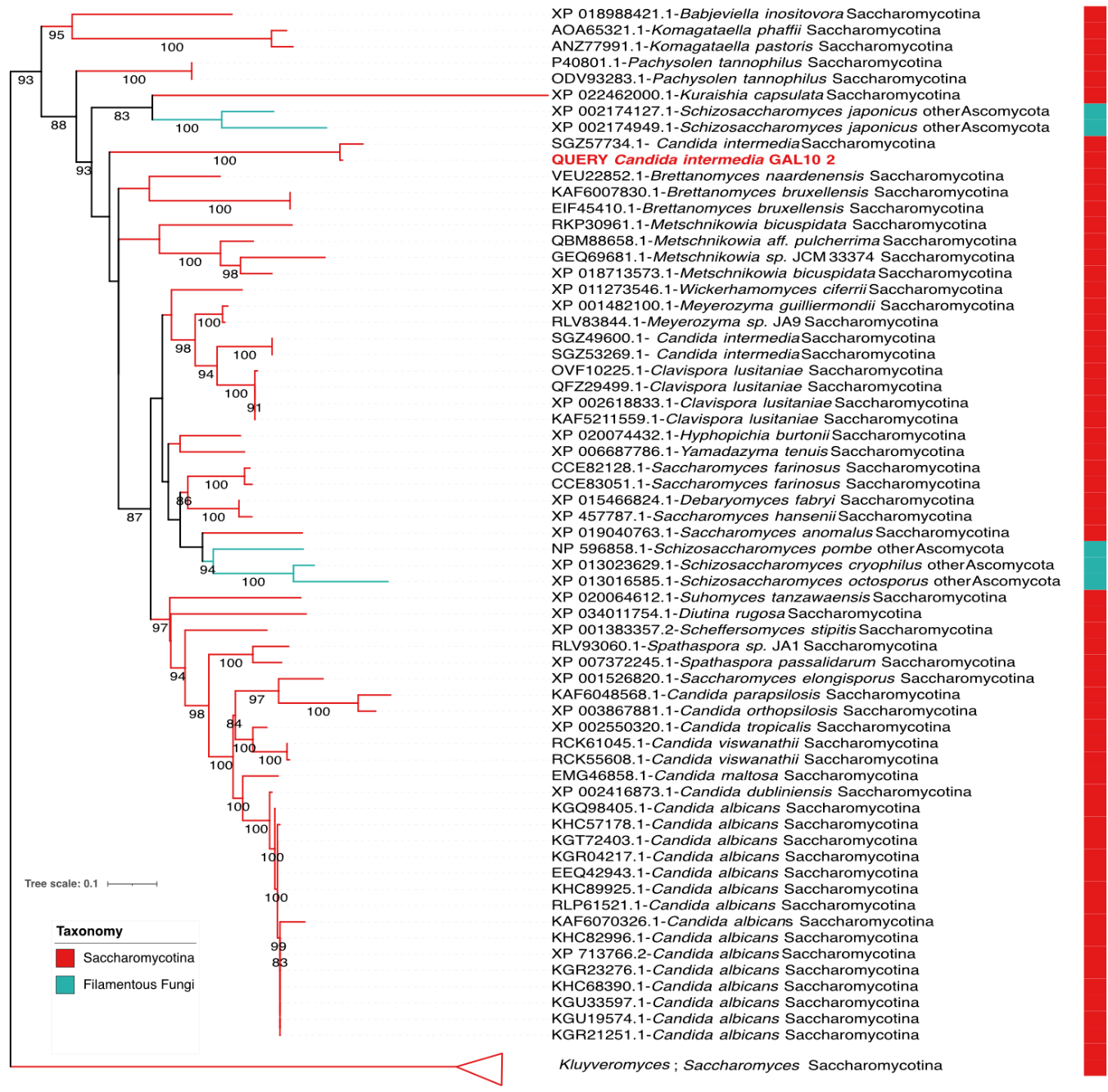


**Figure S4: Maximum likelihood phylogenetic tree for the origin and evolution of the GAL10_2 gene in C. intermedia.**


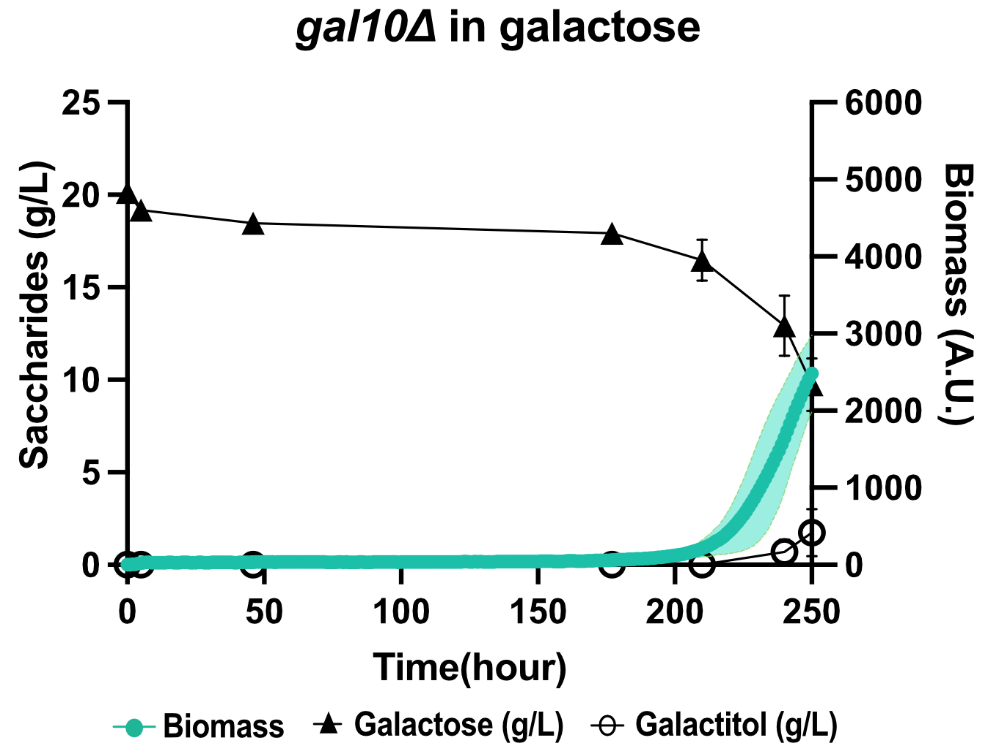


**Figure S5: Growth and metabolite profile for gal10Δ in galactose containing minimal media**. Graph represents biomass (filled green circle) on the right y-axis, consumption of respective sugars (filled triangle for galactose in g/L) and metabolite production (open circle for galactitol in g/L) on the left y-axis, plotted against time (in hours) on x axis. Data are represented as mean ± standard deviation for biological triplicates.


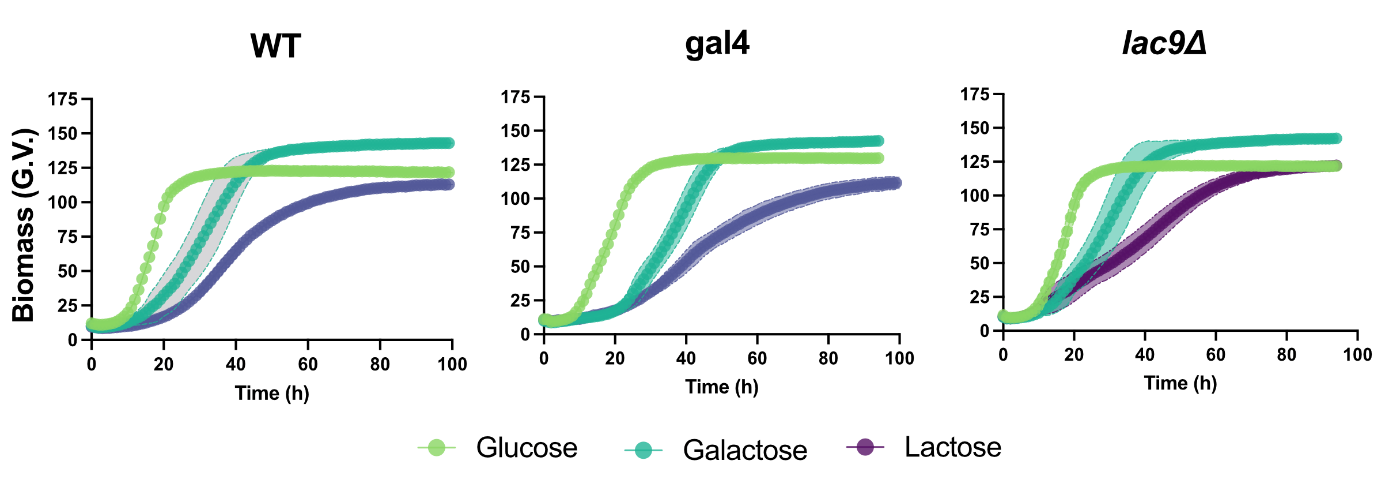


**Figure S6: Growth profiles for WT, gal4Δ and lac9Δ mutants** in glucose (light green), galactose (dark green) and lactose (purple) containing media. Time (in hours) on x-axis is plotted against biomass yield (green values – G.V.) on y-axis. Data are represented as mean ± standard deviation for biological triplicates.
